# Carbon based functional materials enable multifunctional flexible strain sensors for wearable and implantable applications

**DOI:** 10.1186/s11671-026-04734-z

**Published:** 2026-07-02

**Authors:** Siqi Wang, Xuemeng Li, Enci Xie, Shuo Gao

**Affiliations:** School of Instrumentation and Optoelectronic Engineering, Beihang University, Beijing, China

**Keywords:** Multifunctional wearable sensors, Implantable bioelectronics, Flexible and biointegrated materials, Novel device architectures

## Abstract

Flexible strain sensors are essential components for wearable electronics, implantable biointerfaces, and soft human–machine systems. As application scenarios expand from epidermal monitoring toward long-term in vivo operation, increasingly stringent requirements are imposed on multifunctionality, mechanical compliance, signal stability, and biointegration. Carbon-based functional materials, owing to their tunable electrical properties, structural versatility, and favorable electromechanical compatibility, have emerged as a central materials platform for next-generation flexible strain sensors. This review presents a comprehensive, mechanism-oriented overview of carbon-enabled flexible strain sensing, encompassing piezoresistive, capacitive, and piezoelectric transduction modes. This review systematically examine how carbon material dimensionality including low-dimensional nanofillers, two-dimensional sheets, and three-dimensional porous, governing sensitivity, durability, and long-term device reliability. Particular emphasis is placed on contrasting the fundamentally different design requirements of wearable and implantable systems, including sensitivity-stability trade-offs, tissue-level mechanical matching, and operational robustness in complex biological environments. Distinct from prior material- or device-centric reviews, this review establishes a unified framework linking carbon architectures, sensing mechanisms, and application contexts, thereby clarifying critical bottlenecks and design principles for advancing multifunctional, biointegrated strain sensors toward practical and translational use.

## Introduction

Flexible strain sensors have emerged as indispensable components in next-generation wearable electronics, implantable biointerfaces, and soft robotic systems [1–3]. By converting mechanical deformation into electrical signals, these devices enable real-time monitoring of human motion, physiological activities, and biomechanical processes across multiple length scales, from subtle skin strain and pulse waves to organ-level deformation in vivo [4, 5]. The rapid expansion of personalized healthcare, human–machine interaction, and intelligent prosthetics has accelerated the demand for strain sensors that are not only highly sensitive and stretchable, but also mechanically compliant, multifunctional, and capable of long-term stable operation in complex biological environments [6].

Conventional rigid sensing platforms, typically based on silicon or metallic thin films, struggle to accommodate large deformation and fail to provide mechanical matching with soft tissues [7, 8]. This mechanical mismatch often results in signal instability, interfacial delamination, and limited durability, particularly in implantable settings [9–11]. Consequently, flexible and stretchable sensing systems built upon elastomers, hydrogels, and textile substrates have gained significant attention [12]. Within these systems, the functional conductive component plays a decisive role in determining electromechanical coupling efficiency, sensitivity-range balance, cyclic stability, and environmental robustness [13, 14]. Among various material platforms explored to date, carbon-based functional materials have demonstrated unique advantages arising from their diverse dimensionality, tunable electronic structures, chemical stability, and structural adaptability [15, 16].

Carbon materials span a wide range of architectures, including zero- and one-dimensional nanofillers, two-dimensional nanosheets, and three-dimensional porous [17, 18]. These materials can construct conductive pathways through point, line, sheet, or spatially interconnected skeletons, enabling distinct electromechanical transduction mechanisms under deformation [19]. Their intrinsic conductivity, high aspect ratio, surface modifiability, and compatibility with polymeric matrices make them particularly suitable for forming deformable conductive networks that respond sensitively to strain while maintaining structural integrity [20]. Moreover, carbon-based systems can be engineered to support multifunctional integration, including self-powered sensing, electrophysiological recording, electrochemical detection, and even mechanical energy harvesting [21].

Despite these advances, several fundamental challenges remain. First, the trade-off between sensitivity and mechanical robustness persists across different sensing mechanisms, particularly near percolation thresholds or under large strain conditions [22]. Second, long-term signal stability in humid, temperature-variable, or biofluid-rich environments remains insufficiently understood, especially for implantable applications [21, 23]. Third, although numerous studies report high-performance devices, scalability, batch-to-batch reproducibility, and translational feasibility are often underexplored [24]. Importantly, wearable and implantable systems impose fundamentally different constraints: while wearable devices prioritize stretchability, conformability, and user comfort, implantable sensors require stringent biocompatibility, interfacial stability, and reliable operation over extended periods in vivo [25]. These distinctions necessitate a systematic framework that links material dimensionality, sensing mechanism, and application context.

**Fig. 1:**
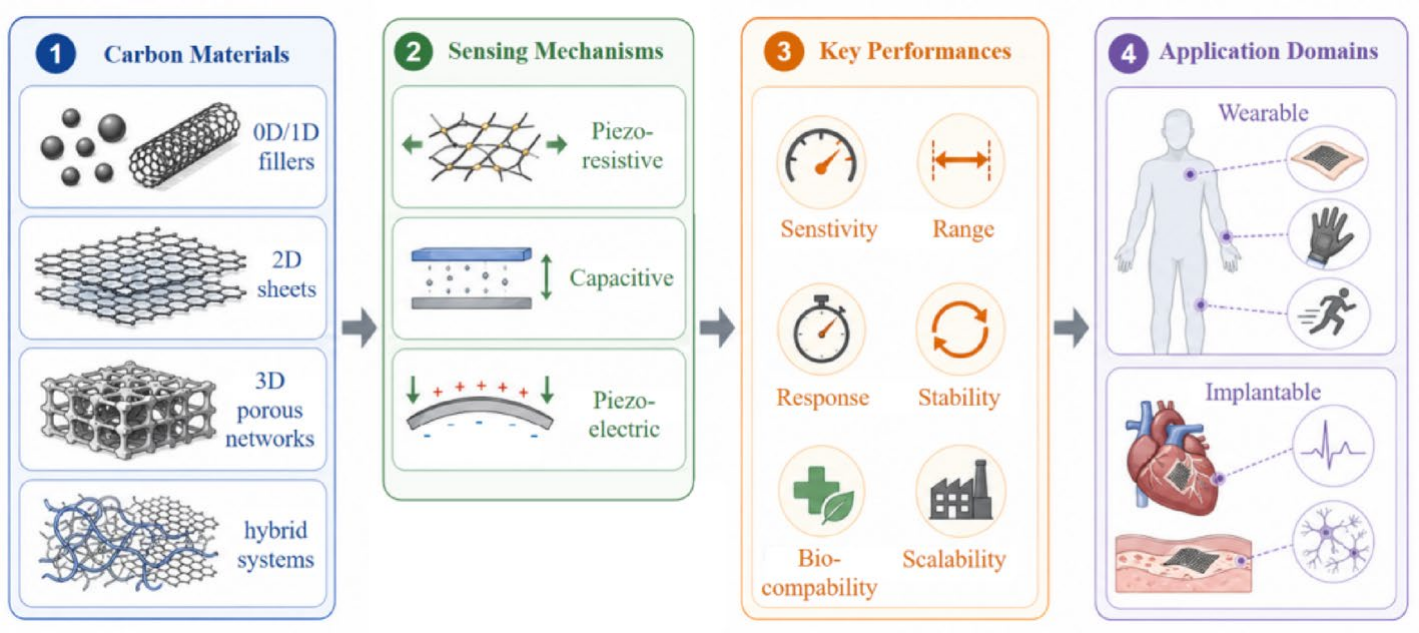
Schematic overview of the review framework

This review provides a comprehensive and mechanism-oriented perspective on carbon-based functional materials enabling multifunctional wearable and implantable flexible strain sensors. We first summarize the fundamental sensing mechanisms including piezoresistive, capacitive, and piezoelectric, and also analyze their electromechanical principles and performance limitations. We then examine how carbon materials of different dimensionalities govern conductive network formation, deformation response, and long-term reliability. Particular emphasis is placed on distinguishing design logic between wearable and implantable systems, highlighting sensitivity-stability trade-offs, mechanical matching strategies, and bioenvironmental adaptability. By establishing a unified structure-mechanism-application framework, this review aims to clarify shared bottlenecks, identify rational design principles, and outline future directions for advancing carbon-enabled strain sensing toward robust, multifunctional, and clinically translatable biointegrated systems. Figure 1 provides a schematic overview of the review framework, summarizing the relationships among carbon material architectures, sensing mechanisms, performance metrics, and wearable/implantable applications. This structure-mechanism-performance-application framework guides the discussion throughout this review.

## Flexible strain sensing mechanism

**Fig. 2:**
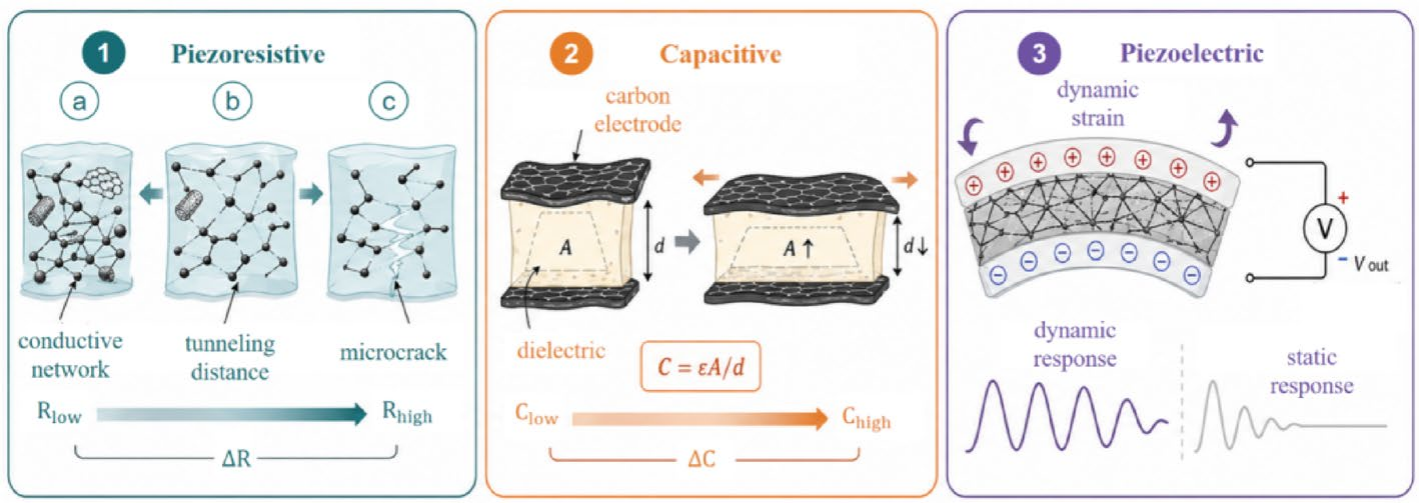
Sensing mechanisms discussed in this review

The sensing mechanism determines how mechanical deformation is converted into measurable electrical signals and therefore plays a central role in defining the sensitivity, linearity, response speed, power requirement, and long-term stability of flexible strain sensors. For carbon-based systems, the transduction behavior is closely related to the deformation-induced evolution of conductive networks, electrode/dielectric geometries, or charge distribution within functional composites. As schematically illustrated in Fig. 2, the representative sensing mechanisms discussed in this review include piezoresistive, capacitive, and piezoelectric mechanisms, which respectively rely on conductive network reconstruction, geometry-induced capacitance variation, and strain-induced charge generation. Understanding these distinct working principles is essential for rationally matching carbon material architectures with wearable and implantable sensing requirements.

### Piezoresistive strain sensing

**Fig. 3:**
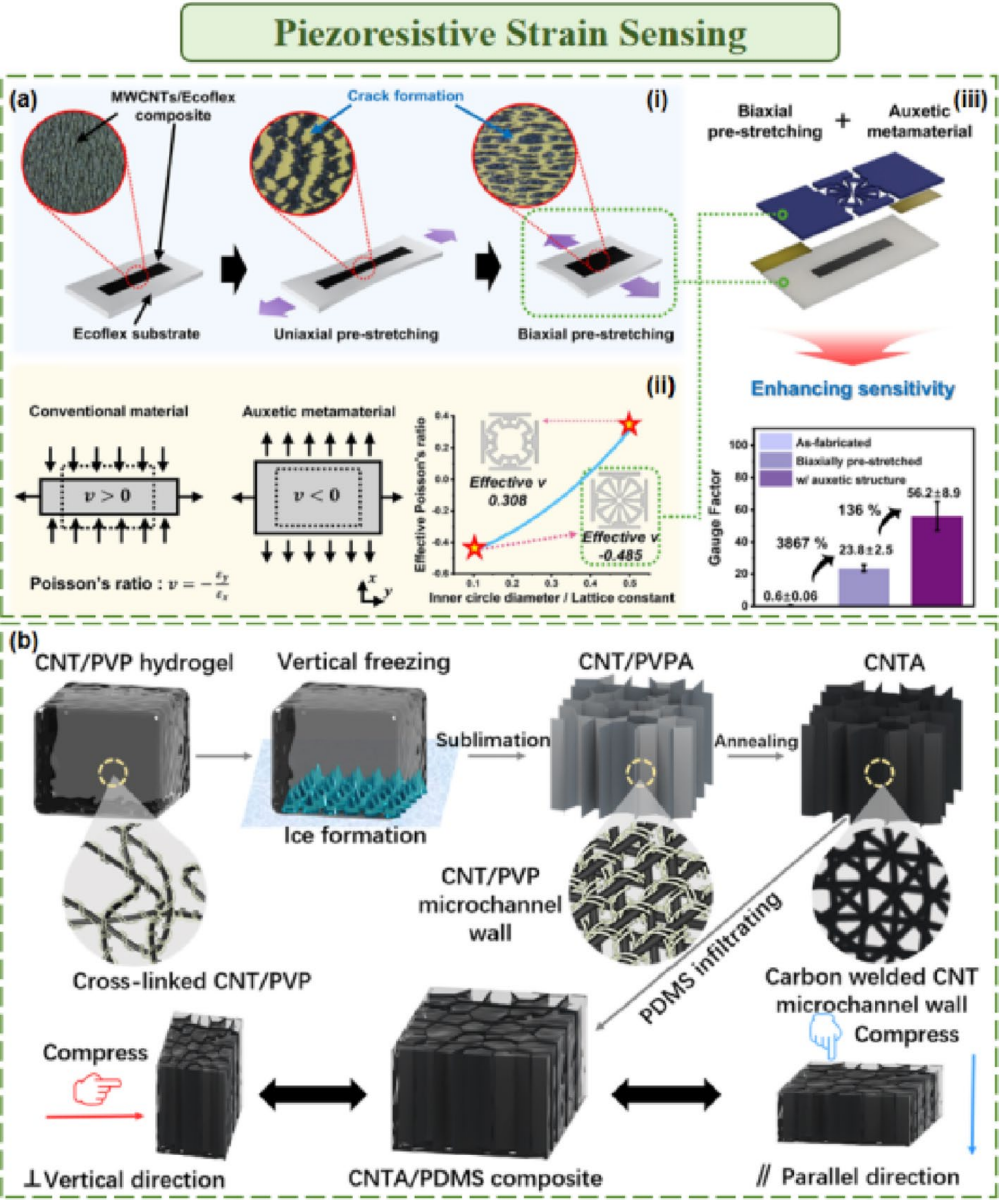
Research status of carbon pressure sensors based on piezoresistive effect. **a** Synergistic microcrack engineering and auxetic metamaterials for stretchable strain sensors with enhanced sensitivity: (i) Crack nucleation driven by biaxial pre-stretching, (ii) Auxetic metamaterials featuring tunable equivalent Poisson's ratio, (iii) A strategy for sensitivity enhancement based on biaxial pre-stretching and auxetic metamaterials. Copyright© 2025 Advanced Materials Technologies published by Wiley‐VCH GmbH; **b** CNTA/PDMS composite fabrication process. Copyright © 2024 Elsevier Ltd

Piezoresistive strain sensing is one of the most extensively adopted transduction strategies in flexible carbon-based sensors because it converts mechanical deformation directly into resistance variation through a relatively simple device configuration. In carbon-enabled piezoresistive systems, the resistance response is primarily governed by deformation-induced reconstruction of conductive networks, including changes in conductive pathway geometry, filler-filler contact resistance, electron tunneling distance, and microcrack evolution [26–28]. When carbon fillers are incorporated near the percolation threshold, small structural perturbations can produce pronounced changes in charge transport pathways, thereby enabling high sensitivity. Similarly, crack-based and tunneling-dominated architectures can amplify resistance variation under subtle strain. As shown in Fig. 3a, Cho et al. enhanced the gauge factor of a MWCNT/Ecoflex composite strain sensor from 15.3 ± 2.1 to 23.8 ± 2.5 by introducing biaxial pre-strain-induced crack structures [29]. This result illustrates a central feature of piezoresistive sensors: their sensitivity is not determined solely by the intrinsic conductivity of carbon materials, but by how effectively the conductive network is engineered to undergo controlled, reversible disruption under deformation.

Despite their structural simplicity and high sensitivity, piezoresistive sensors are intrinsically governed by a trade-off between signal amplification and network reliability. Strategies that maximize gauge factor often rely on operating close to the percolation threshold, enlarging tunneling gaps, or promoting microcrack propagation [30, 31]. These mechanisms enhance the resistance response under small deformation, but they also increase susceptibility to nonlinearity, hysteresis, irreversible network rearrangement, and baseline drift under repeated or large strain [32–35]. Therefore, an ultrahigh gauge factor should not be interpreted as a sufficient indicator of device superiority. For practical wearable and implantable applications, sensitivity must be evaluated together with working range, cyclic stability, response/recovery behavior, hysteresis, environmental robustness, and device-to-device reproducibility. This consideration is particularly important for carbon-based composites, where small variations in filler dispersion, interfacial bonding, and local crack morphology can lead to substantial differences in electromechanical output.

The application logic of piezoresistive sensing differs markedly between wearable and implantable systems. In wearable devices, piezoresistive sensors are attractive because they allow straightforward resistance readout, low-power operation, and facile integration with elastomers, textiles, porous substrates, and skin-conformal platforms [35–38]. These features make them suitable for human motion monitoring, tactile sensing, pulse detection, and soft robotic interfaces. However, wearable operation also exposes the sensing layer to sweat, humidity, temperature variation, repeated attachment-detachment, and motion artifacts, all of which can accelerate resistance drift or weaken calibration reliability. In implantable settings, the challenge becomes more stringent: the sensor must maintain stable conductive pathways in biofluid-rich, mechanically dynamic, and biologically active environments without causing mechanical irritation or inflammatory responses. Thus, for implantable piezoresistive sensors, long-term signal stability, encapsulation reliability, tissue-level mechanical matching, and prevention of conductive filler leakage are often more critical than achieving the highest initial sensitivity.

Recent advances have attempted to mitigate these limitations through anisotropic network design, porous architectures, microstructure regulation, and interfacial protection. As shown in Fig. 3b, Li et al. developed anisotropic carbon nanotube aerogel/PDMS composites that retained the deformation-sensitive conductive network of CNT aerogels while enabling direction-dependent strain responses [39]. Gao et al. introduced wrinkle-based microstructures through polymer swelling and chemical deposition, offering another route to tune local deformation and resistance modulation [40]. Such strategies demonstrate that piezoresistive performance can be improved not simply by increasing carbon filler content, but by rationally designing the deformation pathway of the conductive network. Nevertheless, many high-performance designs still depend on narrowly controlled microstructures or multi-step fabrication processes, which raises concerns about scalability, batch-to-batch consistency, and long-term reliability. Future development should therefore shift from pursuing maximum sensitivity toward reliability-centered network engineering, including reversible crack control, stable filler-matrix interfaces, drift-resistant encapsulation, and standardized evaluation under physiologically relevant mechanical and environmental conditions.

Piezoresistive carbon-based strain sensors offer a compelling balance of high sensitivity, simple readout, and broad material compatibility, making them especially powerful for flexible and wearable sensing. However, their practical value is ultimately determined by whether the conductive network can remain reversibly deformable and electrically stable over long-term operation. For next-generation wearable and implantable systems, the key design question is not how to obtain the largest resistance change, but how to engineer a carbon network that produces a sufficiently sensitive, repeatable, and biologically reliable signal under realistic use conditions.

### Capacitive strain sensing

Capacitive strain sensing is another important transduction strategy in flexible carbon-based sensors, in which mechanical deformation is converted into capacitance variation through changes in device geometry or dielectric properties. For typical parallel-plate or quasi-parallel-plate configurations, the capacitance is determined by the effective electrode area, interelectrode distance, and dielectric constant of the insulating layer [41–43]. Under stretching, compression, or bending, deformation alters these parameters and produces a measurable capacitive response. Compared with piezoresistive sensing, capacitive sensing is more strongly governed by macroscopic structural deformation rather than by the disruption of microscopic conductive pathways. Therefore, its sensitivity is usually limited by geometric constraints, and a single capacitive unit rarely achieves an ultrahigh gauge factor [44]. However, this mechanism also gives capacitive sensors important advantages, including relatively good linearity, low hysteresis, and reduced baseline drift, making them particularly attractive for long-term and continuous monitoring applications [45, 46].

**Fig. 4:**
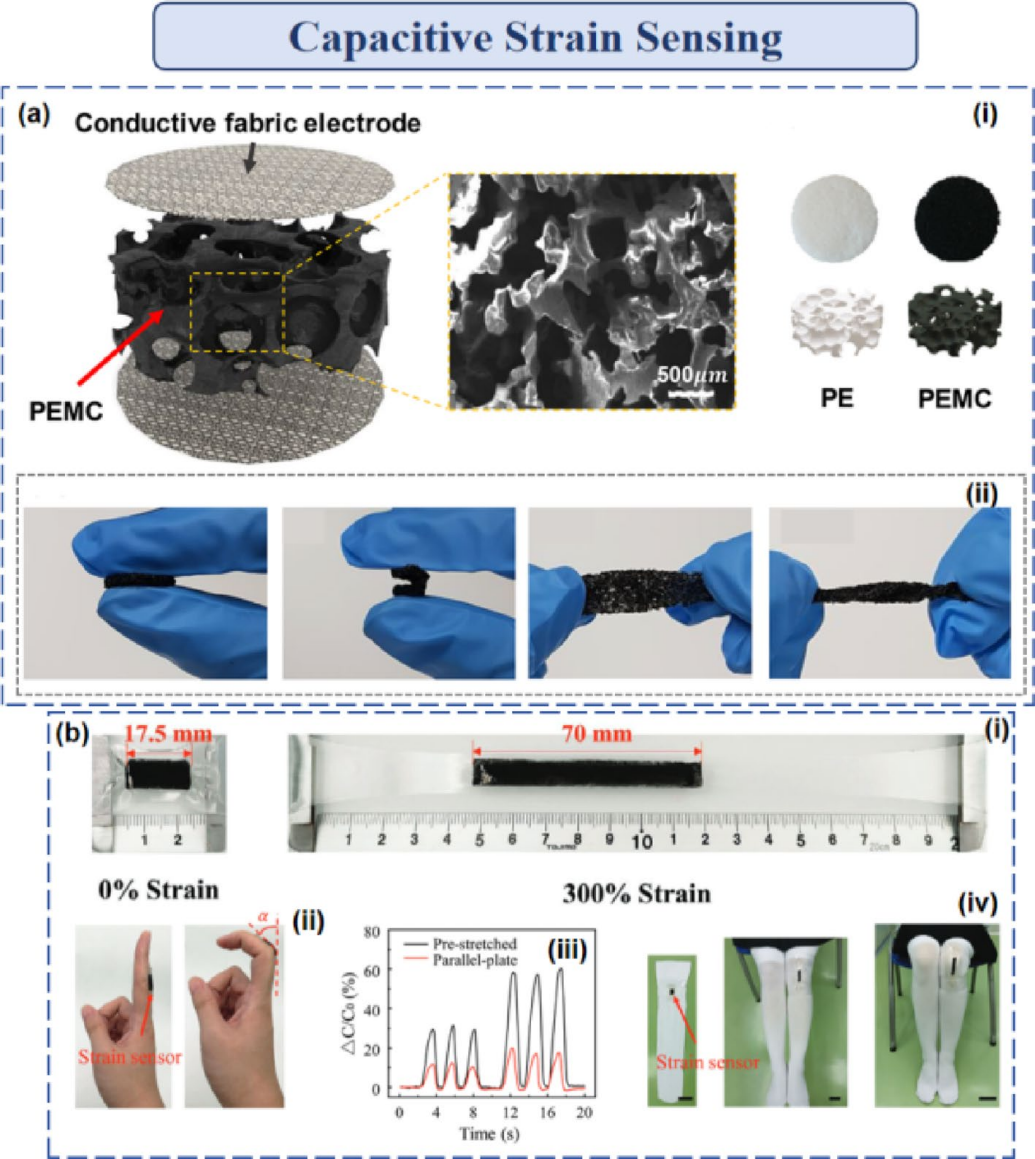
Research status of capacitive carbon pressure sensors. **a** Performance capacitive pressure sensors based on high porous elastomer and percolation of carbon nanotube filler: (i) Structure of porous Ecoflex-multiwalled carbon nanotube composite (PEMC), (ii) Photos of the sensor's deformation under force. Copyright © 2020, American Chemical Society; **b** Super-stretchable and highly sensitive carbon nanotube capacitive strain sensor. (i) Stretchablility of the capacitive strain sensor, (ii) Test on fingure, (iii) Strain responses under thefinger bending and relaxing cycles at the three pose, (iv) Test on knee. Copyright © 2021 Wiley‐VCH GmbH

The performance of carbon-based capacitive sensors depends critically on the mechanical deformability and electrical stability of the electrodes and dielectric layer. Carbon materials, especially CNTs, graphene, and porous carbon composites, are commonly used as flexible electrodes or conductive fillers because they can maintain electrical continuity during large deformation while preserving mechanical compliance. In this context, porous and wrinkled structures are widely employed to amplify deformation-induced capacitance changes. As shown in Fig. 4a, Choi et al. developed a porous Ecoflex/multi-walled carbon nanotube composite capacitive pressure sensor with an improved sensitivity of 6.42 kPa^−1^ [47]. The porous elastomeric structure enhanced compressibility and increased the effective deformation of the dielectric layer, thereby improving pressure response while maintaining softness. Similarly, as shown in Fig. 4b, Hu et al. reported an ultrastretchable capacitive strain sensor composed of two wrinkled CNT electrodes separated by a tape dielectric layer, achieving a gauge factor of 2.07 at 300% strain with high linearity and negligible hysteresis [48]. These examples indicate that the key to enhancing capacitive sensing performance is not merely increasing electrode conductivity, but designing deformable electrode-dielectric architectures that can translate external strain into stable and reversible capacitance changes.

Despite their favorable stability, capacitive strain sensors face several system-level challenges that are fundamentally different from those of piezoresistive sensors. First, capacitance variations are often relatively small, especially under low strain or miniaturized device dimensions, requiring high-resolution readout circuits and careful noise suppression [49–51]. Second, capacitive signals are susceptible to parasitic capacitance, electromagnetic interference, cable motion, and environmental coupling, which may become more pronounced in wearable and implantable platforms with flexible interconnects and compact circuit layouts. Third, although the sensing mechanism itself can exhibit low hysteresis, long-term reliability still depends on the mechanical integrity of the dielectric layer, electrode adhesion, and encapsulation stability [52, 53]. In this sense, capacitive sensors shift the central design challenge from conductive-network stability to device-structure and circuit-level stability.

The application advantages of capacitive sensors are therefore most evident in scenarios where stable, repeatable, and low-drift signals are more important than extremely high sensitivity. In wearable systems, capacitive sensors are well suited for posture monitoring, joint motion tracking, tactile sensing, and long-duration health monitoring because their relatively linear output and low hysteresis facilitate calibration and signal interpretation. However, practical wearable use still requires suppression of parasitic capacitance caused by body proximity, sweat, clothing friction, and motion-induced cable deformation. In implantable systems, capacitive architectures are attractive because they can operate with mechanically compliant electrodes and soft dielectric materials, potentially enabling stable monitoring of tissue deformation. Yan et al. developed an implantable direct bladder interface system using low-modulus silicone elastomers, CNTs, platinum-PDMS nanocomposites, and medical-grade wires, integrating strain sensing with bladder muscle electrical stimulation [54]. Nevertheless, implantable capacitive sensors must further address biofluid insulation, dielectric aging, encapsulation reliability, and low-power readout, since even small leakage currents or interfacial degradation can compromise signal accuracy over long-term operation.

Recent research has attempted to improve capacitive sensor performance through porous dielectrics, wrinkled or stretchable electrodes, flexible encapsulation layers, and low-loss dielectric materials [47, 48, 55, 56]. These strategies can extend the working range, improve linearity, and reduce mechanical failure under repeated deformation. However, they also introduce new trade-offs. Highly porous dielectrics may enhance sensitivity but can reduce mechanical durability or increase environmental susceptibility. Wrinkled electrodes improve stretchability but require controlled fabrication to maintain reproducibility. High-precision readout circuits improve resolution but increase system complexity and power consumption, which is especially problematic for implantable or wireless platforms. Therefore, future development of carbon-based capacitive strain sensors should focus on integrated optimization of material architecture, structural mechanics, circuit design, and encapsulation rather than on sensitivity enhancement alone.

Capacitive carbon-based strain sensors should be viewed as stability-oriented sensing platforms. Their main strength lies in linear, low-hysteresis, and drift-resistant signal acquisition, while their limitations arise from relatively small signal amplitude and strong dependence on device geometry and readout electronics. For wearable and implantable applications, the most important design goal is not to compete directly with piezoresistive sensors in gauge factor, but to develop mechanically robust, low-noise, low-power, and long-term stable capacitive systems that can operate reliably under realistic biological and environmental conditions.

### Piezoelectric strain sensing

Piezoelectric strain sensing converts mechanical deformation into electrical signals through strain-induced charge separation or voltage generation in piezoelectric materials. Compared with piezoresistive and capacitive mechanisms, piezoelectric sensing is distinguished by its ability to generate electrical outputs without an external bias, making it attractive for low-power and self-powered flexible sensing systems [57–59]. However, piezoelectric output is intrinsically associated with time-varying deformation. As a result, piezoelectric sensors are highly suitable for detecting dynamic or periodic mechanical signals, such as pulse, respiration, vibration, body movement, and organ motion, but they are generally less effective for quantitative monitoring of static or slowly varying strain because the output signal tends to decay over time [60]. Therefore, piezoelectric strain sensing should be regarded as a dynamic-event-oriented mechanism rather than a universal solution for all deformation-monitoring scenarios.

**Fig. 5:**
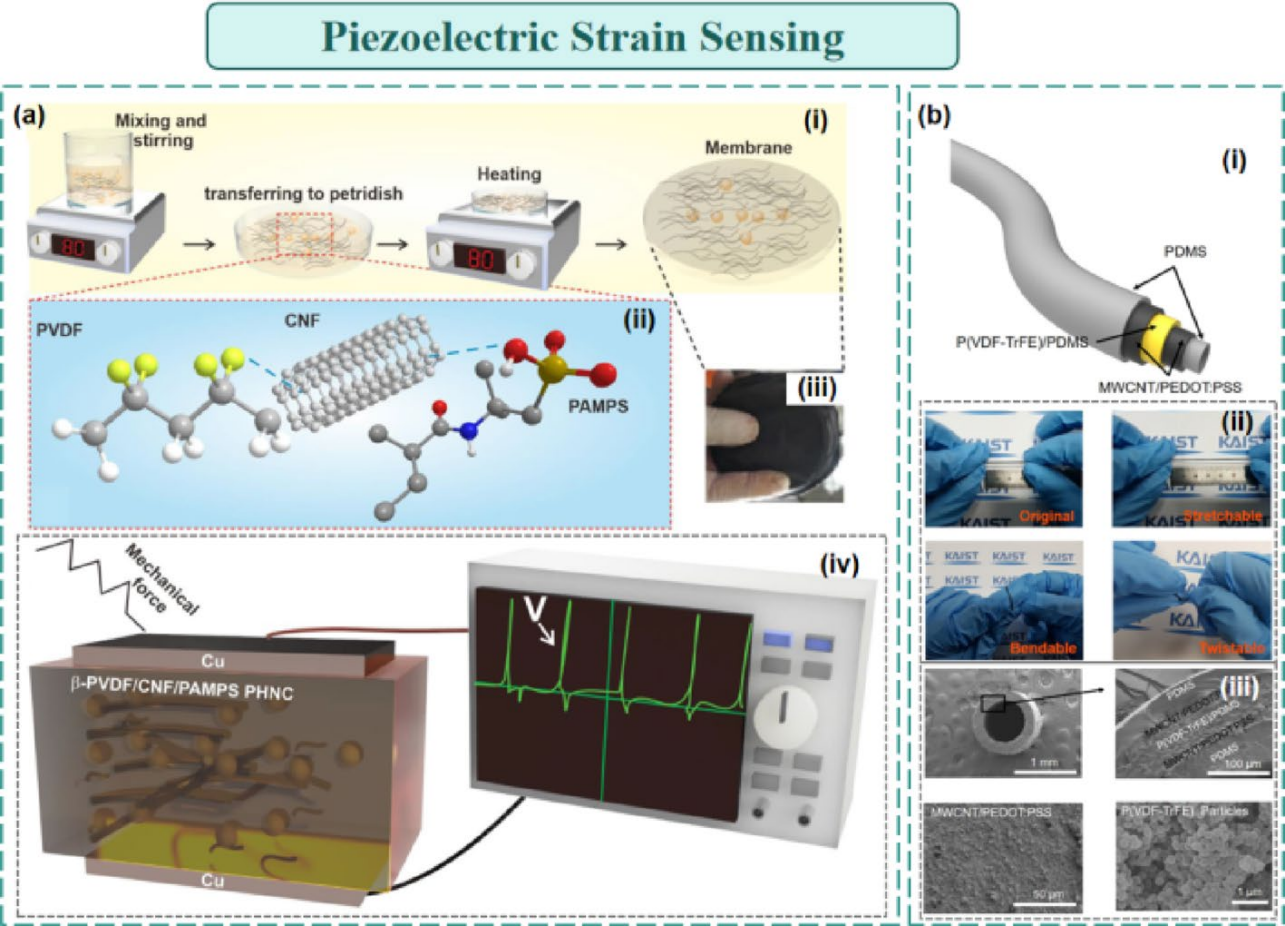
Research status of carbon pressure sensors based on piezoelectric effect. **a** Hydrophilic piezoelectric nanocomposite via CNF/polyelectrolyte triggering for high voltage sensing: (i) Preparation process of piezoelectric membrane, (ii) Structure of PVDF, CNF, and PAMPS, (iii) Piezoelectric membrane, (iv) Schematic diagram of the test process. Copyright © 2022 Elsevier B.V. **b** (i) Schematic illustration of SMF fiber, (ii) Photos of SMF fiber deformation, (iii) SEM image of SMF fiber cross section. Copyright © 2018 Elsevier Ltd

In carbon-enabled piezoelectric systems, carbon materials usually play multiple roles rather than acting as conventional piezoelectric phases alone. Carbon nanotubes, carbon nanofibers, graphene derivatives, and carbon-based porous structures can enhance electrical conductivity, facilitate charge collection, improve stress transfer, promote piezoelectric phase formation in polymer matrices, or construct mechanically compliant pathways for deformation transmission [61–64]. For example, carbon nanofibers and nanotubes have been widely incorporated into poly(vinylidene fluoride) (PVDF)-based composites to promote the formation of the polar β-phase and improve electromechanical output. As shown in Fig. 5a, Panwar et al. introduced hydrophilic poly(2-acrylamido-2-methylpropane sulfonic acid) into PVDF/carbon nanofiber composites, inducing microstructural rearrangement and enhancing β-phase formation, which increased the peak output voltage to 3.65 V [65]. This example illustrates that carbon materials can improve piezoelectric sensing performance not only by increasing conductivity, but also by regulating polymer crystallization, interfacial polarization, and mechanical stress transfer.

Carbon-based piezoelectric systems also provide opportunities for wearable and implantable sensing where low-power operation and dynamic physiological monitoring are desired [66, 67]. Li et al. reported a pronounced piezoelectric response in high-surface-area carbon nanotube yarns coated with an electrolyte layer, where tensile deformation generated an axial voltage gradient without external electrical bias [68]. Such ionic or interfacial piezoelectric effects expand the functional role of carbon materials beyond passive electrodes and suggest potential applications in smart textiles, wearable sensing, and artificial muscle feedback. In addition, flexible piezoelectric structures can be coupled with energy-harvesting functions, enabling event-triggered sensing or auxiliary power supply. As shown in Fig. 5b, Ryu et al. developed an intrinsically stretchable multifunctional hollow-fiber architecture that enabled strain sensing and mechanical energy harvesting through pressure generated by internal fluid flow [69]. These examples suggest that piezoelectric carbon-based devices are particularly suitable for transient, periodic, and mechanically active environments.

Nevertheless, the practical use of piezoelectric strain sensors is constrained by several mechanism-specific limitations. First, because piezoelectric signals are generated mainly under dynamic deformation, static strain, posture maintenance, or slow tissue deformation cannot be reliably monitored without additional signal-processing strategies or hybrid sensing mechanisms [59, 70]. Second, the output amplitude is strongly affected by polarization state, strain rate, stress transfer efficiency, electrode contact, and environmental conditions such as temperature and humidity [71–74]. Third, in soft and stretchable devices, mechanical energy may be dissipated by the elastomeric matrix or hydrogel substrate before it is effectively transferred to the piezoelectric phase, resulting in attenuated output and reduced reproducibility. These limitations indicate that piezoelectric performance should not be evaluated solely by peak voltage or instantaneous output, but should also include signal stability, frequency response, mechanical durability, calibration reliability, and output consistency under repeated deformation.

The design requirements of piezoelectric sensors also differ substantially between wearable and implantable systems. In wearable applications, piezoelectric sensors are advantageous for detecting pulse waves, respiration, gait-induced vibration, joint motion, and other dynamic biomechanical signals. Their self-generated electrical output can reduce external power demand and support lightweight sensing systems. However, wearable operation introduces motion artifacts, inconsistent contact pressure, sweat-induced interface changes, and variations in sensor placement, all of which can influence output amplitude and waveform interpretation. In implantable systems, piezoelectric sensing may provide a promising route for monitoring cardiac beating, vascular pulsation, respiratory motion, gastrointestinal peristalsis, or other periodic organ deformation. However, long-term implantation imposes more stringent requirements on biocompatibility, stable encapsulation, fatigue resistance, and mechanical matching with soft tissues. In addition, biofluid exposure may alter interfacial polarization, electrode stability, and dielectric behavior, making long-term signal calibration a major challenge.

Recent efforts have addressed these issues through material modification, phase engineering, flexible structural design, and system-level co-design. Incorporating carbon nanofibers, carbon nanotubes, or graphene derivatives into piezoelectric polymers can improve β-phase formation, charge transport, and mechanical reinforcement [65]. Hydrogel-based and glycerol-modified systems can broaden environmental tolerance and maintain signal output under variable temperature or humidity conditions [75]. Hollow fibers, porous structures, and stretchable architectures further improve mechanical compliance and enable coupling between sensing and energy harvesting [69]. However, many reported devices are still evaluated under controlled laboratory conditions, and their long-term performance under realistic wearable or implantable environments remains insufficiently established. Future development should therefore focus on hybrid sensing architectures, stable encapsulation, frequency-dependent calibration, fatigue-resistant interfaces, and standardized evaluation protocols that distinguish dynamic sensing capability from true long-term deformation monitoring.

Piezoelectric carbon-based strain sensors are best understood as self-powered or low-power dynamic sensing platforms. Their greatest strength lies in detecting transient, periodic, and vibration-like mechanical events, whereas their main limitation lies in weak static strain quantification and strong dependence on deformation frequency, interfacial polarization, and mechanical energy transfer. For next-generation wearable and implantable applications, piezoelectric sensors may be most effective when integrated with piezoresistive or capacitive elements, allowing dynamic event detection to be combined with stable static or quasi-static strain monitoring..

Although piezoresistive, capacitive, and piezoelectric strain sensors can all be constructed using carbon-based functional materials, their signal transduction principles, device configurations, and application suitability differ substantially. Therefore, a direct comparison of these mechanisms is necessary for understanding how carbon materials should be selected and engineered for different wearable and implantable scenarios. Table 1 summarizes the representative sensing mechanisms discussed in this review, with emphasis on their working principles, commonly used carbon-based materials, major advantages, and intrinsic limitations.

**Table 1:** Comparison of representative sensing mechanisms in carbon-based flexible strain sensors

Mechanism	Signal transduction principle	Typical carbon-based materials	Key advantages	Main limitations
Piezoresistive sensing	Mechanical deformation induces changes in conductive pathways, contact resistance, tunneling distance, and/or microcrack evolution, leading to resistance variation	CNTs, MWCNTs, carbon black, graphene, rGO, CNT aerogels, carbon nanofibers	Simple device structure; direct resistance readout; high sensitivity; easy integration with elastomers, textiles, hydrogels, and porous substrates	Nonlinearity, hysteresis, signal drift, environmental sensitivity, and sensitivity-strain range trade-off, especially near the percolation threshold
Capacitive sensing	Strain changes electrode area, electrode spacing, and/or dielectric layer geometry, causing capacitance variation	CNT electrodes, graphene electrodes, porous CNT/elastomer composites, carbon-based flexible electrodes	Good linearity; relatively low hysteresis; stable cyclic response; suitable for long-term continuous monitoring	Lower intrinsic sensitivity; small capacitance variation; susceptible to parasitic capacitance and electromagnetic interference; requires precise readout circuits
Piezoelectric sensing	Dynamic mechanical deformation induces charge or voltage output through piezoelectric or piezoionic effects	CNT yarns, carbon nanofiber/PVDF composites, CNT hydrogels, carbon-assisted piezoelectric polymer composites	Self-powered signal generation; high signal-to-noise ratio for dynamic or periodic deformation; suitable for low-power systems	Poor response to static or quasi-static strain; signal decay over time; sensitive to polarization state, humidity, and temperature

As shown in Table 1, no single sensing mechanism can simultaneously satisfy all requirements of high sensitivity, wide sensing range, long-term stability, low power consumption, and implantable compatibility. Piezoresistive sensors are attractive because of their simple structure and high sensitivity, but they often suffer from hysteresis and signal drift caused by irreversible conductive network reconstruction. Capacitive sensors generally provide better linearity and cyclic stability, yet their relatively small signal variation requires more sophisticated readout circuits. Piezoelectric sensors offer advantages for dynamic and low-power sensing, but their outputs are strongly dependent on time-varying deformation and environmental stability. Therefore, mechanism selection should be guided by the target application rather than by a single performance metric.

## Carbon-based materials for wearable and implantable sensing

**Fig. 6:**
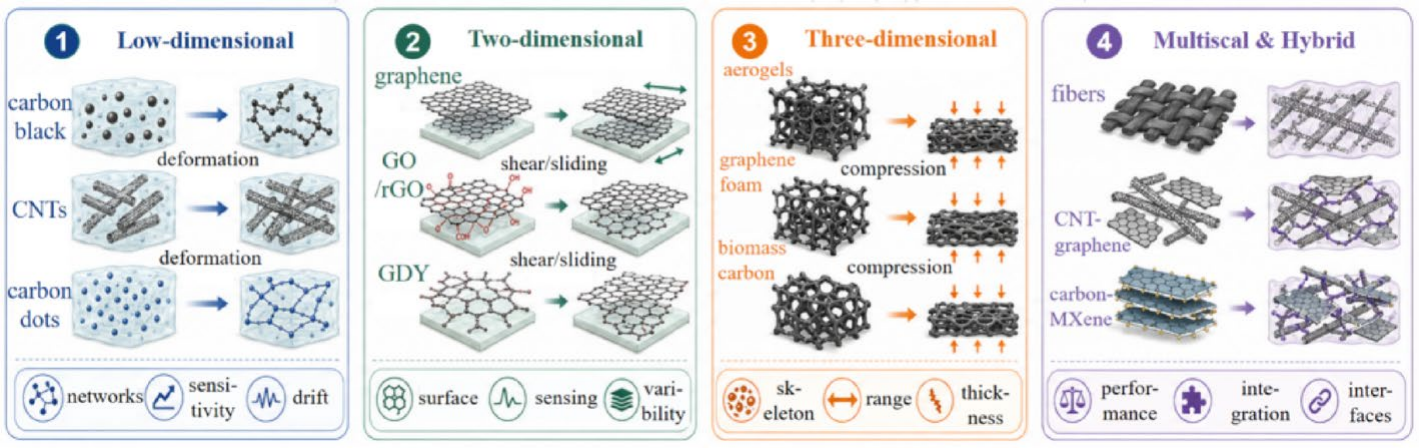
Categorized carbon-based sensing materials

Carbon-based functional materials serve as the core active components that determine the electromechanical response, mechanical compliance, environmental stability, and integration capability of flexible strain sensors. Their sensing behavior is strongly governed by material dimensionality and architecture, because carbon particles, nanotubes, nanosheets, porous networks, and hybrid systems form conductive pathways with distinct deformation responses. As illustrated in Fig. 6, representative carbon-based sensing materials can be broadly categorized into low-dimensional carbon nanofillers, two-dimensional carbon materials, three-dimensional porous architectures, and multiscale hybrid systems, each showing different structure–property-application relationships. These material classes differ in conductive network formation, deformation tolerance, and application-oriented suitability for wearable and implantable systems. Therefore, this section discusses carbon-based sensing materials according to their structural dimensionality and highlights how carbon architectures should be rationally selected for different sensing requirements.

### Low-dimensional carbon nanofillers

Low-dimensional carbon nanofillers, including conductive carbon black, carbon nanotubes, carbon nanofibers, and carbon dots, represent one of the most widely used material families for constructing deformable conductive networks in flexible strain sensors [76–80]. Their small feature size, large specific surface area, and compatibility with elastomers, hydrogels, and textile substrates allow efficient electromechanical coupling under stretching, bending, compression, and subtle biological deformation. In these systems, the sensing response is mainly governed by deformation-induced changes in percolative pathways, contact resistance, and tunneling distance between adjacent conductive units. However, these fillers should not be regarded as interchangeable conductive additives. Their aspect ratio, intrinsic conductivity, dispersibility, and interfacial stability lead to distinct performance boundaries and application-dependent trade-offs.

**Fig. 7:**
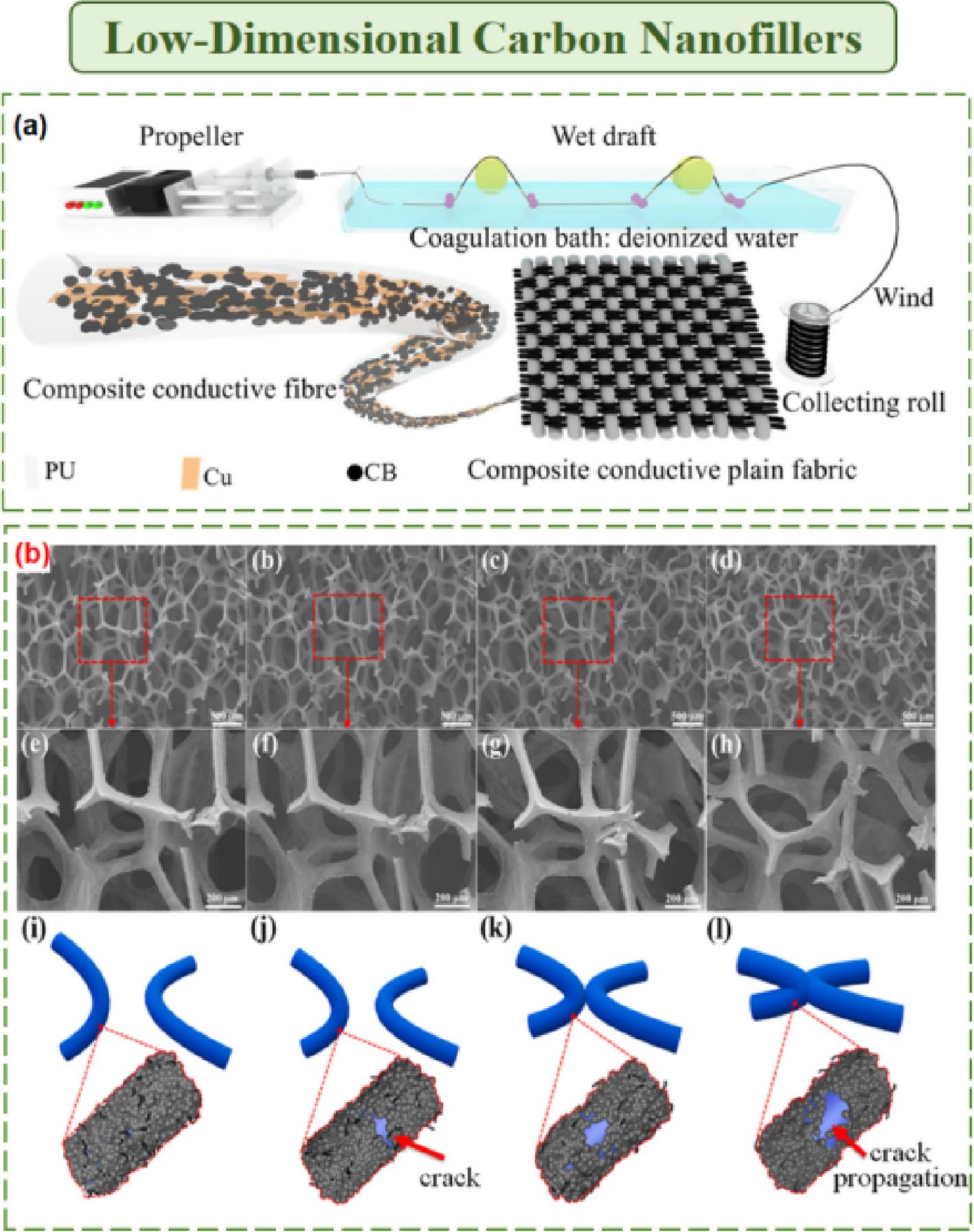
Representative flexible strain sensors based on low-dimensional carbon nanofillers. **a** Schematic diagram of the stretchable conductive fibers, plain fabric, and internal distribution of fibers. Copyright © 2025 The Authors. Published by Elsevier B.V. **b** SEM images of conductive PU sponge and schematic of the sponge skeleton and conductive network with different compression strain. Copyright © 2021 Elsevier B.V

Conductive carbon black is among the earliest and most mature conductive fillers used in flexible strain and pressure sensors. Although its intrinsic conductivity is lower than that of carbon nanotubes or graphene, carbon black offers clear engineering advantages, including low cost, simple processing, stable particle morphology, and good compatibility with scalable manufacturing routes [81–83]. These features make it particularly suitable for large-area wearable sensors, elastic fibers, and textile-integrated sensing platforms, where mechanical durability and manufacturing simplicity are often more important than achieving an ultrahigh gauge factor. For example, carbon black-containing stretchable conductive fibers can be woven or integrated into fabric-based sensing systems, providing a practical route for wearable motion monitoring and textile electronics (Fig. 7a). The sensing response of carbon black-based composites is typically dominated by contact resistance variation among neighboring particles. This mechanism can provide robust and repeatable signals under moderate deformation, but it also imposes intrinsic limitations on sensitivity and detection resolution. Because carbon black particles have low aspect ratios, relatively high filler loading is usually required to establish a continuous conductive network, which may increase the composite modulus and compromise softness, stretchability, and tissue-level mechanical matching [84]. Therefore, carbon black is better suited for mechanically robust and scalable wearable sensing systems than for highly sensitive or long-term implantable strain sensors.

Carbon nanotubes and carbon nanofibers provide a different design logic. Their high aspect ratios and excellent electrical conductivity allow percolated networks to form at much lower filler concentrations, thereby preserving the compliance of elastomeric or hydrogel matrices while maintaining high electrical sensitivity [85–89]. Under deformation, one-dimensional conductive units can slide, rotate, reconnect, or separate within the matrix, generating pronounced resistance changes while delaying complete network failure. This feature explains why CNT- and CNF-based sensors are often advantageous for stretchable strain sensing, soft robotic perception, and wearable motion monitoring [90–94]. Representative CNT-based architectures, such as CNT-coated porous scaffolds and CNT-coated elastic fabrics, have demonstrated high compressibility, rapid response, and good cyclic durability in flexible sensing systems [95–98]. In particular, CNT-coated polyurethane sponge scaffolds provide interconnected conductive pathways along the porous skeleton, allowing resistance variation through skeleton compression and contact reconstruction under external loading (Fig. 7b). Nevertheless, the same network reconfigurability that enables high sensitivity also introduces reliability concerns. CNTs tend to agglomerate because of strong van der Waals interactions, leading to spatially nonuniform conductive pathways and device-to-device variability. During repeated large deformation or long-term operation, irreversible rearrangement of nanotube contacts may cause hysteresis, baseline drift, and sensitivity degradation. These problems are particularly important for implantable systems, where recalibration is difficult and long-term signal stability is often more critical than peak initial sensitivity.

To improve the stability of CNT-based sensors, current strategies mainly focus on surface functionalization, polymer encapsulation, alignment control, and the construction of hybrid conductive networks. For example, combining CNTs with carbon black or other conductive fillers can lower the percolation threshold, improve response uniformity, and partially compensate for the limitations of single-filler systems [86]. Crack-bridging designs based on CNT-coated fabrics can also help maintain electrical continuity during deformation [98]. However, these strategies should be viewed critically. Enhanced sensitivity or stretchability is often achieved by introducing more complex microstructures and additional interfaces, which may increase fabrication difficulty and create new failure pathways. Therefore, CNT-based flexible strain sensors should not be evaluated only by gauge factor or maximum strain range. More systematic reporting of hysteresis, long-term cyclic drift, inter-batch variation, filler migration, encapsulation reliability, and biological response is needed, especially when implantable applications are claimed.

Carbon dots have recently expanded the functional scope of low-dimensional carbon materials. Unlike carbon black and CNTs, carbon dots are not primarily used to construct highly conductive percolation networks. Their value lies in tunable fluorescence, electrochemical activity, rich surface functional groups, and generally favorable biocompatibility [99–101]. These properties make them promising for multimodal sensing systems that combine mechanical sensing with optical, electrochemical, or biochemical readouts. In hydrogel-based wearable or implantable interfaces, carbon dots can contribute to biofunctionalization, signal amplification, ion/electron transport modulation, or biochemical responsiveness. However, their relatively low intrinsic electrical conductivity limits their use as the main conductive phase in strain sensors. In most cases, carbon dots must be combined with CNTs, carbon black, graphene, or conductive polymers to achieve sufficient electrical output [102–105]. Such hybridization improves multifunctionality but also complicates material design, signal calibration, and long-term reproducibility. Therefore, carbon dots are more appropriately considered as functional modifiers or multimodal sensing components rather than standalone strain-sensing fillers.

The different requirements of wearable and implantable sensors further reshape the evaluation of low-dimensional carbon nanofillers. In wearable systems, key priorities include low-cost fabrication, large-area integration, washability, comfort, and stable signal output during repeated body motion. Carbon black- and CNT-based composites are therefore attractive for smart textiles, skin-mounted sensors, and motion monitoring platforms. In implantable systems, however, the design priorities shift toward low modulus, minimal foreign-body response, long-term biofluid stability, reliable encapsulation, and resistance to signal drift. Low filler loading is desirable to reduce mechanical mismatch and potential biological risks, but insufficient filler content may weaken electrical stability. This creates a central design conflict: percolation-sensitive networks provide high strain sensitivity, but they are also vulnerable to irreversible reconstruction in long-term physiological environments.

Low-dimensional carbon nanofillers provide a mature and versatile foundation for flexible strain sensors, but their practical value depends strongly on application context. Carbon black offers robustness, low cost, and scalability; CNTs and CNFs provide high sensitivity and stretchability; carbon dots introduce multimodal and biofunctional possibilities [106, 107]. The key challenge is no longer simply to identify a more conductive filler, but to engineer conductive networks that remain mechanically compliant, electrically stable, biologically safe, and reproducible over prolonged operation. For wearable devices, future development should balance sensitivity with comfort, durability, and scalable processing. For implantable devices, progress will depend on reliable encapsulation, suppression of filler migration, long-term drift compensation, and systematic in vivo validation. These considerations suggest that low-dimensional carbon nanofillers should be designed not as isolated performance enhancers, but as components of an integrated material-interface-device system.

### Two-dimensional carbon-based materials

Two-dimensional carbon-based materials, represented by graphene, graphene oxide (GO), reduced graphene oxide (rGO), and emerging graphdiyne (GDY), provide sheet-like conductive building blocks for flexible strain sensors [108–110]. Compared with zero-dimensional carbon particles and one-dimensional nanotubes, two-dimensional nanosheets can form extended in-plane conductive pathways and large-area interfacial contacts within elastomers, hydrogels, or flexible substrates. Their sensing response is mainly governed by deformation-induced changes in intersheet contact area, tunneling distance, sheet sliding, wrinkle evolution, and microcrack propagation. These features make 2D carbon materials particularly attractive for high-resolution detection of small strain, pressure, and tactile stimuli. However, their practical use is strongly limited by sheet restacking, interfacial instability, processing variability, and irreversible structural rearrangement during repeated deformation.

**Fig. 8:**
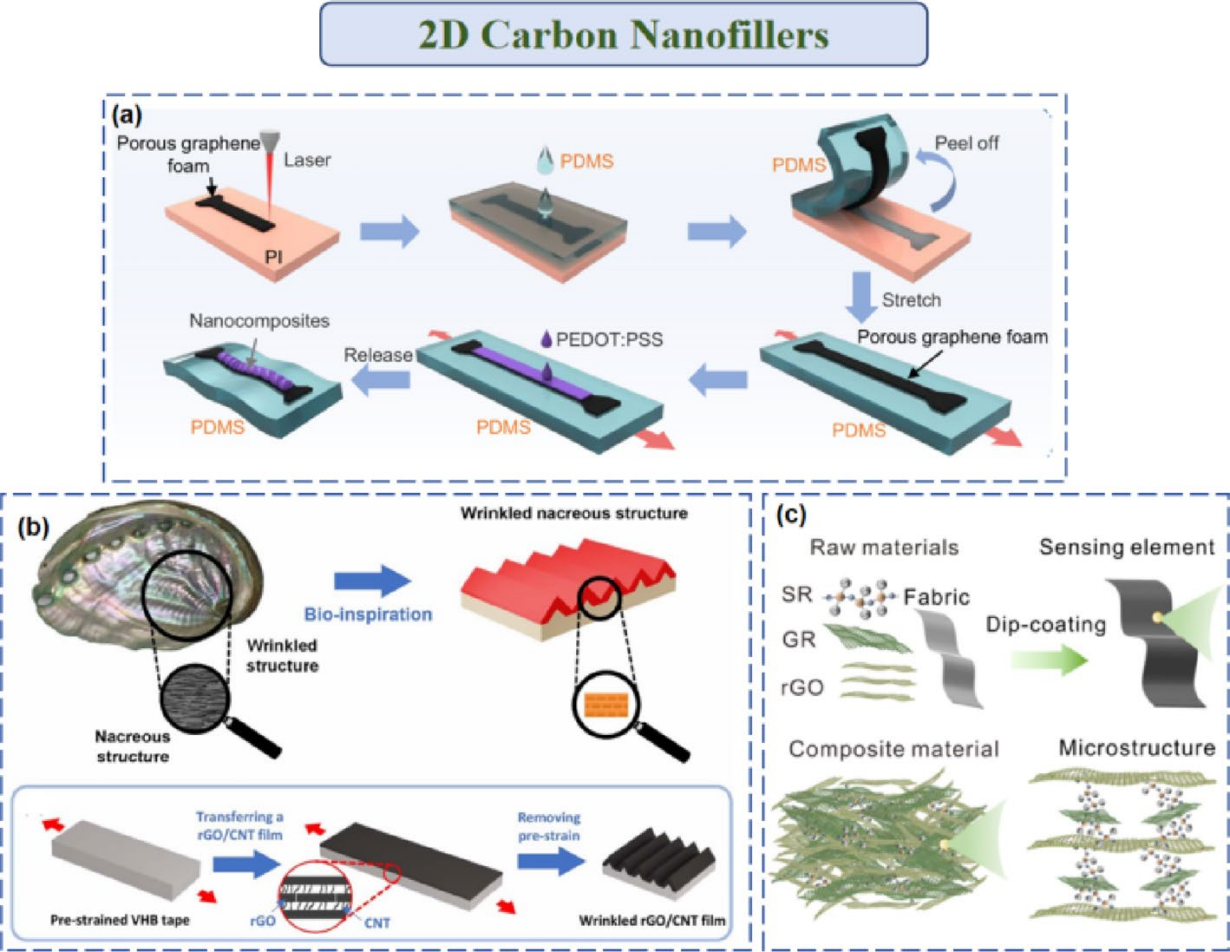
Strain sensor with 2D-dimensional carbon nanofillers. **a** Fabrication of graphene-based strain sensor. Copyright © 2025 Springer Nature. **b** Schematic of the rGO/CNT film and its fabrication on a stretchable adhesive film. Copyright © 2024 All authors. **c** Substrate selection process and the microstructure within the conductive network. Copyright © 2025 Elsevier B.V

Graphene possesses high intrinsic electrical conductivity, high carrier mobility, and excellent mechanical strength, enabling sensitive electromechanical transduction at relatively low filler loadings [111]. In graphene-based strain sensors, even subtle deformation can alter the contact state between adjacent nanosheets, leading to pronounced resistance changes. For example, graphene nanoplatelet-based sensing layers on flexible substrates can exhibit very high gauge factors under small strain, illustrating the strong electromechanical coupling enabled by sheet-like conductive networks (Fig. 8a) [112]. Nevertheless, the same structural sensitivity that enables ultrahigh gauge factors also creates reliability concerns. Graphene sheets are prone to sliding, overlapping, and restacking because of strong van der Waals interactions. Under large strain or prolonged cyclic loading, these rearrangements may become partially irreversible, causing hysteresis, baseline drift, and degradation of sensitivity [113]. Therefore, graphene-based sensors are generally more suitable for high-resolution and small-to-moderate strain detection than for applications requiring extreme stretchability or long-term mechanical cycling without recalibration.

GO and rGO are widely used to address the processability limitations of pristine graphene. The oxygen-containing functional groups on GO improve hydrophilicity, dispersibility, and interfacial adhesion with polymer or hydrogel matrices, which facilitates the fabrication of uniform flexible composites [114, 115]. However, these functional groups disrupt the conjugated carbon network and significantly reduce electrical conductivity. Partial reduction to rGO restores part of the conductivity while retaining acceptable dispersibility, making rGO one of the most practical 2D carbon materials for flexible strain sensors. A representative strategy is the construction of rGO/CNT hybrid “brick-and-mortar” structures, in which rGO sheets provide broad conductive interfaces while CNTs bridge neighboring sheets and suppress complete network disconnection during deformation (Fig. 8b) [116]. This type of hybrid design reflects an important principle for 2D carbon sensors: high sensitivity should not rely solely on sheet separation or crack opening, but should be balanced by conductive bridges or elastic interfaces that maintain network recoverability.

Despite these advantages, the performance of GO/rGO-based sensors remains highly dependent on processing conditions. The degree of oxidation or reduction, defect density, sheet size distribution, residual functional groups, and interfacial bonding state can all affect electrical conductivity and mechanical reliability [117]. As a result, devices with similar material names may exhibit substantially different gauge factors, strain ranges, hysteresis, and cyclic stability. This issue is particularly important for review-level evaluation because many reported improvements arise from narrowly optimized microstructures rather than universally transferable material advantages. For practical wearable and implantable systems, the key challenge is not only to achieve high sensitivity, but also to ensure batch-to-batch reproducibility, stable interfacial adhesion, and predictable signal output under long-term deformation.

In wearable sensing systems, graphene and rGO-based composites are most valuable for detecting subtle deformation, tactile stimuli, and low-pressure signals, where their high surface area and sheet-contact sensitivity can be fully exploited. For instance, rGO-based flexible networks have been used for posture monitoring, sleep monitoring, and warning of abnormal body movements (Fig. 8c) [118]. However, wearable applications also expose 2D carbon networks to repeated bending, stretching, sweat, humidity, and mechanical abrasion. Without appropriate encapsulation or interface design, sheet sliding and microcrack evolution can gradually alter the conductive network, leading to drift and reduced repeatability. Therefore, future wearable sensors based on 2D carbon materials should report not only peak sensitivity, but also long-term cycling stability, washing resistance, humidity tolerance, and sensor-to-sensor variation.

For implantable sensors, the evaluation criteria become even stricter. Graphene and rGO are attractive for flexible electrodes, bioelectronic interfaces, and electrochemical sensing because their sheet dimensions and surface chemistry can be tuned [119, 120]. However, implantable strain sensing requires more than electrical conductivity and flexibility. Long-term exposure to biofluids may alter sheet interfaces, weaken adhesion to soft matrices, and induce electrochemical side reactions associated with defects, edges, or residual oxygen-containing groups. In addition, detached nanosheets or insufficiently encapsulated conductive layers may raise biosafety concerns. Therefore, in implantable applications, 2D carbon materials are more suitable as encapsulated conductive interfaces or functional coatings than as directly exposed sensing networks. Their translational potential depends on stable surface modification, reliable encapsulation, tissue-level mechanical matching, and long-term in vivo validation.

Graphdiyne has recently emerged as a new member of the 2D carbon family. Unlike graphene, GDY contains both sp- and sp^2^-hybridized carbon atoms and possesses an intrinsically porous structure, which may provide additional ion transport channels, molecular adsorption sites, and electrochemical activity [121–123]. These features make GDY conceptually attractive for multimodal sensing and biointerface applications. However, compared with graphene and rGO, GDY-based flexible strain sensors are still at an early stage. The preparation of high-quality GDY generally involves more complex synthesis and transfer processes, and the availability, reproducibility, mechanical robustness, and long-term stability of GDY-based devices remain insufficiently demonstrated. Therefore, GDY should currently be regarded as a promising but immature 2D carbon platform rather than a near-term replacement for graphene or rGO in wearable and implantable strain sensors.

Two-dimensional carbon-based materials offer high sensitivity, large interfacial contact area, and strong potential for multifunctional biointerfaces, but their performance advantages are accompanied by clear structural and processing constraints. Graphene provides excellent conductivity and sensitivity but suffers from restacking and irreversible sheet rearrangement. GO/rGO improves processability and interfacial compatibility but introduces variability associated with reduction degree and defect chemistry [124–126]. GDY offers new opportunities for electrochemical and multimodal sensing but remains limited by synthesis complexity and immature device validation [127]. For wearable applications, 2D carbon materials are most suitable for high-resolution detection of subtle strain and tactile signals. For implantable applications, their future use will depend on stable encapsulation, controlled surface chemistry, suppression of electrochemical side reactions, and systematic evaluation of long-term biointerface stability [128–131]. Thus, the key design principle for 2D carbon-based flexible sensors is to convert their high interfacial sensitivity into reproducible and durable device-level performance, rather than merely pursuing ultrahigh initial gauge factors.

### Three-dimensional porous and networked carbon architectures

Compared with low-dimensional fillers that form conductive pathways through discrete particles, nanotubes, or nanosheets, three-dimensional porous and networked carbon architectures establish electrical conduction through a continuously interconnected spatial skeleton [132]. Typical examples include carbon aerogels, graphene foams, 3D graphene networks, and biomass-derived porous carbon structures. Their high porosity, low density, large internal surface area, and compressible frameworks enable efficient conversion of pressure, compression, and large-scale deformation into electrical signals. In these materials, the sensing response is mainly governed by pore collapse, skeleton contact reconstruction, crack evolution, and changes in electron transport pathways within the interconnected carbon framework [133, 134]. Therefore, 3D carbon architectures are particularly attractive for pressure sensing, tactile perception, and large-area wearable sensing systems. However, their relatively large thickness, structural heterogeneity, and fabrication complexity also limit their use in ultrathin skin-conformal devices and long-term implantable interfaces.

**Fig. 9:**
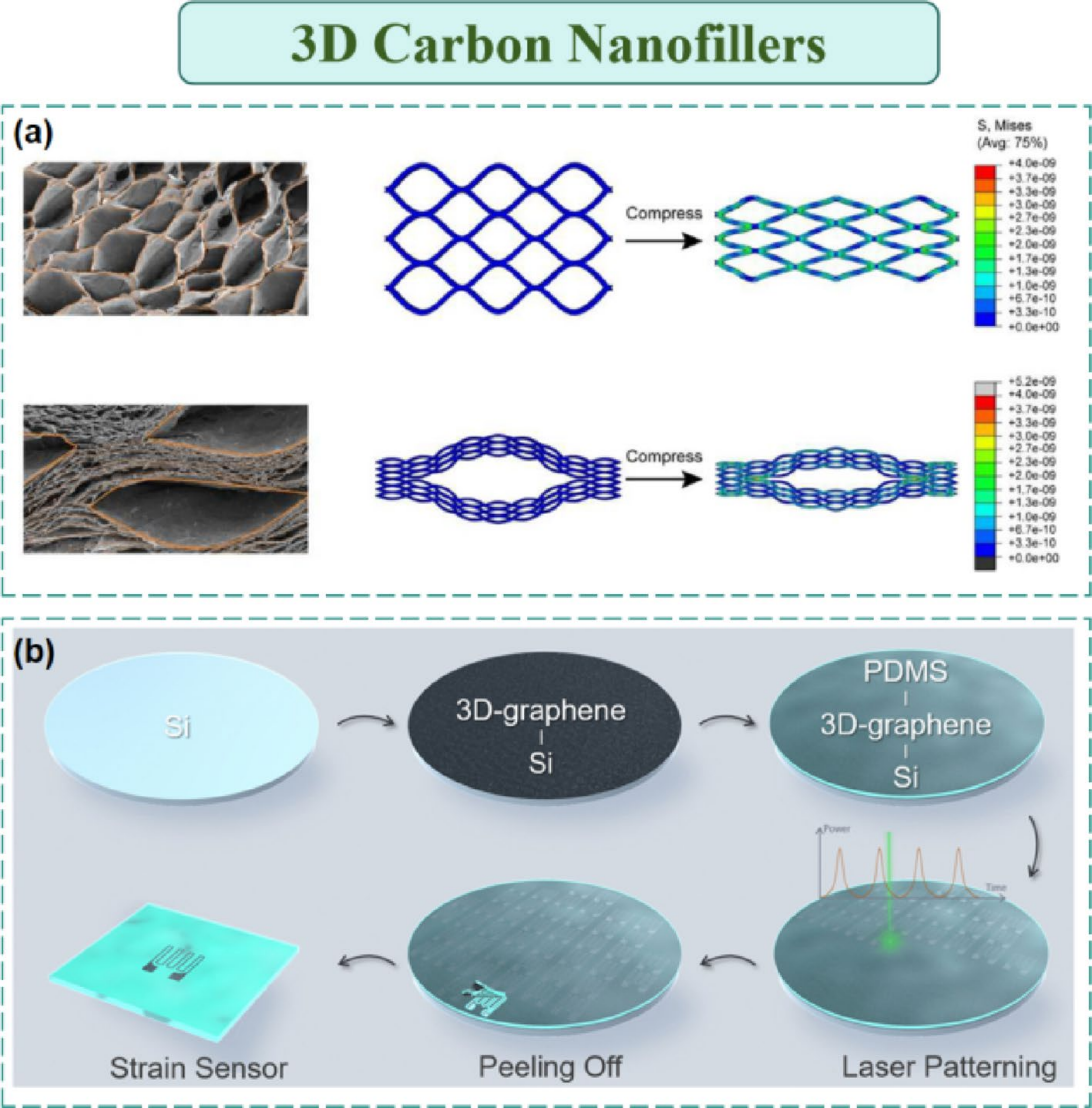
Strain sensor with 3D-dimensional carbon nanofillers. **a** Finite element (FE) analysis of stress distribution in models under strain. Copyright © 2022 Elsevier Ltd. **b** Fabrication and characterization of 3D-graphene flexible sensors. Copyright © 2025 All authors

Carbon aerogels are representative 3D porous carbon materials composed of continuous nanoscale carbon skeletons and highly tunable pore networks [76, 77]. Under external compression, their porous structures undergo reversible deformation, leading to changes in skeleton contact and conductive pathways. This mechanism allows carbon aerogel-based sensors to exhibit high pressure sensitivity and broad working ranges, especially under compressive or tactile loading. For example, graphene aerogels with hierarchical or honeycomb-like porous structures have been used to detect physiological pressure signals by amplifying local deformation through their cellular framework (Fig. 9a) [135]. Compared with conventional composite sensors based on dispersed fillers, aerogel-based sensors reduce the uncertainty associated with filler dispersion because the carbon skeleton itself serves as the main sensing network. Nevertheless, this advantage is accompanied by mechanical fragility. Unreinforced carbon aerogels are prone to skeleton fracture, pore collapse, or irreversible deformation under repeated compression and bending. Polymer infiltration, elastomer coating, and hybrid aerogel construction can improve toughness and recoverability, but these strategies may also reduce porosity, alter sensitivity, and introduce additional interfacial failure modes [136]. Thus, the key challenge for carbon aerogel sensors is to preserve deformation-sensitive porous networks while preventing irreversible structural damage during long-term cyclic operation.

Three-dimensional graphene networks and graphene foams provide another important class of 3D carbon architectures. By assembling graphene sheets into a continuous spatial framework, these materials combine the high conductivity of graphene with the deformability of porous structures [78, 79]. Their electromechanical response is primarily determined by changes in intersheet contact, local crack formation, and framework deformation under strain or compression. Compared with randomly dispersed graphene nanosheets, 3D graphene networks can provide more stable conductive pathways and reduce restacking-induced loss of active surface area. For instance, wafer-scale 3D graphene structures fabricated on silicon substrates and patterned into flexible devices have shown anisotropic electromechanical responses and improved sensitivity under directional strain (Fig. 9b) [20]. However, 3D graphene structures often require template-assisted growth, chemical reduction, or laser processing, making their structural uniformity and batch-to-batch reproducibility difficult to control. In addition, although their porous frameworks improve deformability, some 3D graphene networks still possess higher effective stiffness than soft biological tissues. This may cause mechanical mismatch in skin-mounted or implantable applications unless they are integrated with soft elastomers, hydrogels, or ultrathin device architectures.

Biomass-derived carbon materials have attracted increasing attention because natural biological templates can provide hierarchical porous structures after carbonization [137]. Wood, cellulose, plant tissues, and other biomass precursors can preserve multiscale channels, aligned pores, or cellular frameworks, offering a low-cost and potentially sustainable route to 3D conductive networks. These structures are advantageous for pressure sensing and large-deformation monitoring because their natural porosity can enhance compressibility and mechanical recovery. However, the same template-dependent structure also introduces a major limitation: the microstructure, electrical conductivity, and mechanical response of biomass-derived carbon are strongly influenced by the original biological source and carbonization conditions [138, 139]. As a result, reproducibility and precise structural control remain more challenging than in synthetic 3D carbon frameworks. For high-precision wearable sensing or implantable monitoring, this variability may lead to inconsistent baseline resistance, sensitivity, and long-term stability.

In wearable sensing systems, 3D porous and networked carbon architectures are most suitable for pressure distribution mapping, tactile feedback, plantar pressure monitoring, and large-area flexible sensing platforms [140–142]. Their porous skeletons can accommodate repeated compression and recover their shape, enabling relatively stable signal output under complex mechanical loading. However, they are not universally superior to low-dimensional filler systems. Because their sensing response often relies on macroscopic volume change or skeleton contact reconstruction, their sensitivity to very small tensile strain may be lower than that of crack-based graphene films or percolation-sensitive CNT networks. Their thickness may also reduce skin conformability and make them less suitable for ultrathin epidermal electronics. Therefore, 3D carbon architectures should be selected when wide working range, compressive stability, and mechanical resilience are prioritized, rather than when the primary requirement is ultrathin format or ultrasensitive small-strain detection.

For implantable sensors, the use of 3D porous carbon architectures remains more challenging and should be evaluated cautiously. Their porous structures can reduce effective modulus and potentially improve mechanical matching with soft tissues [143–145]. However, long-term exposure to biofluids may cause pore fouling, encapsulation failure, signal drift, or unwanted tissue ingrowth into open porous networks. These issues are particularly problematic when the porous carbon skeleton is directly exposed to the biological environment. Moreover, the relatively large volume and complex internal structure of 3D porous materials make sterilization, encapsulation, and long-term mechanical reliability more difficult than for thin-film or hydrogel-based interfaces. Therefore, in implantable applications, 3D carbon architectures may be more appropriate as encapsulated conductive scaffolds, compliant electrode supports, or mechanical buffer layers rather than directly exposed sensing interfaces.

Three-dimensional porous and networked carbon architectures provide a distinct sensing strategy based on spatial framework deformation rather than only particle contact, nanotube tunneling, or nanosheet sliding. Carbon aerogels offer high porosity and pressure sensitivity but suffer from fragility and irreversible skeleton damage. Three-dimensional graphene networks provide improved conductivity and framework continuity but face challenges in scalable fabrication, stiffness control, and structural reproducibility. Biomass-derived carbon offers sustainability and naturally hierarchical porosity, but its template-dependent variability limits precision and consistency [146–149]. For wearable devices, these materials are especially valuable for compressive, tactile, and large-area sensing. For implantable devices, future progress will depend on controlling pore architecture, improving mechanical robustness, preventing biofluid-induced degradation, and developing reliable encapsulation strategies. The central design principle is therefore to translate the structural advantages of 3D carbon frameworks into stable, reproducible, and biointerface-compatible device performance, rather than simply exploiting high porosity as a sensitivity-enhancing feature.

### Multiscale and hybrid carbon reinforcement systems

As flexible strain sensors move from single-function devices toward wearable and implantable multifunctional systems, single-scale carbon materials often struggle to simultaneously satisfy high sensitivity, wide working range, mechanical robustness, signal stability, and scalable fabrication. Multiscale and hybrid carbon reinforcement systems have therefore been developed to combine carbon materials with different dimensions, morphologies, and mechanical roles [150, 151]. In these systems, macroscale carbon fibers or fabrics can provide structural integrity, while nanoscale fillers such as carbon nanotubes, carbon black, graphene, or other conductive additives can improve local electromechanical sensitivity. The central advantage of hybrid design is not simply the addition of more conductive components, but the construction of hierarchical conductive pathways that remain responsive under subtle deformation while maintaining electrical continuity under large strain.

Carbon fibers and carbon-based fabrics are typical macroscale skeletons in multiscale carbon sensing systems. Their continuous fibrous structures and high mechanical strength allow them to maintain structural integrity during repeated bending, stretching, and textile-level deformation [152]. After surface functionalization with conductive nanomaterials such as CNTs, graphene, or MXene, carbon fabrics can serve as flexible, large-area sensing platforms for smart textiles and wearable motion monitoring [153]. However, carbon fibers themselves are relatively stiff compared with skin or soft tissues. Direct use of rigid fibrous skeletons may reduce wearing comfort, generate local stress concentration, or cause mechanical mismatch at soft biointerfaces. Therefore, carbon fabrics are more appropriately used as mechanically robust carriers or textile-integrated substrates, rather than as standalone sensing layers for highly compliant or implantable applications.

Hybrid filler networks provide another important strategy for balancing sensitivity and mechanical stability. By combining low-dimensional fillers such as CNTs, carbon black, and graphene, conductive networks can be constructed across multiple length scales [154]. For example, CNTs can bridge separated conductive domains, carbon black can enhance contact resistance modulation, and graphene sheets can provide extended interfacial conduction. Such complementary roles can lower the percolation threshold, improve response uniformity, and expand the working strain range compared with single-filler systems. Nevertheless, hybridization should not be viewed as an automatic route to superior performance. The final sensing behavior depends strongly on filler dispersion, interfacial adhesion, filler-matrix compatibility, and the mechanical evolution of each conductive component under cyclic deformation [155]. Poorly controlled hybrid networks may introduce more failure pathways than single-component systems, including phase separation, local stress accumulation, irreversible conductive pathway reconstruction, and increased device-to-device variation.

MXene/carbon composite systems have recently become an active direction in multiscale sensing because MXenes provide high electrical conductivity and abundant surface functional groups, while carbon materials can improve structural stability, flexibility, and network continuity. In MXene/CNT, MXene/graphene, or MXene/carbon textile systems, carbon components can act as conductive bridges, mechanical supports, or anti-restacking frameworks, whereas MXene layers can contribute high surface conductivity and interfacial interaction. Representative MXene-decorated textiles and MXene/CNT bilayer films have demonstrated sensitive responses to human motion and tunable sensing ranges [156, 157]. However, these systems also illustrate a broader challenge of multifunctional hybrid sensors: enhanced performance often comes with increased material instability and encapsulation demands. MXene is susceptible to oxidation in humid or biofluid-rich environments, and its long-term performance strongly depends on hydrophobic modification, barrier layers, or encapsulation strategies [158]. For implantable applications, this problem becomes more severe because the sensing layer must remain stable under continuous exposure to ions, proteins, tissue motion, and inflammatory responses.

In wearable sensing systems, multiscale and hybrid carbon architectures are particularly useful when the device must respond to both small and large mechanical stimuli. Hierarchical networks can maintain conductive continuity during joint bending, textile deformation, and repeated body motion, while local nanoscale contacts or cracks provide amplified electrical responses to subtle strain. This makes hybrid systems attractive for smart textiles, motion monitoring, electronic skin, and human–machine interfaces [159–161]. However, wearable applications also expose devices to sweat, washing, mechanical abrasion, and repeated attachment-detachment cycles. Therefore, the practical value of hybrid carbon sensors should be evaluated not only by sensitivity or stretchability, but also by washability, environmental stability, long-term drift, comfort, and manufacturing reproducibility.

For implantable systems, multiscale and hybrid carbon architectures require a more cautious evaluation. Although hierarchical structures can reduce the effective modulus and improve mechanical matching with soft tissues, multi-component systems inevitably introduce more interfaces and potential failure sites [162–165]. Each interface, including carbon-polymer, carbon-MXene, filler-hydrogel, and coating-tissue interfaces, may undergo delamination, swelling, corrosion, or biofouling during long-term implantation. In addition, hybrid systems complicate biosafety assessment because different components may exhibit distinct degradation, migration, or inflammatory behaviors. Therefore, multiscale carbon systems are more suitable for use as encapsulated sensing layers, functional integration platforms, or electrode-support structures in implantable devices, rather than as directly exposed biointerfaces [166]. Their translational feasibility depends on reliable encapsulation, minimized material leakage, stable interfacial bonding, and systematic long-term in vivo validation.

Multiscale and hybrid carbon reinforcement systems provide a powerful route for overcoming the limitations of single carbon materials, but their benefits arise from rational structural complementarity rather than simple material stacking. Macroscale carbon fabrics offer mechanical robustness and textile compatibility, nanoscale fillers provide local sensitivity, and MXene or other functional additives can introduce additional electrical or interfacial functions. The major challenge is that every added component also introduces new interfaces, processing variables, and possible failure modes. For wearable devices, future development should focus on durable, washable, scalable, and comfortable hybrid structures. For implantable devices, priority should be given to simplified material compositions, stable encapsulation, biointerface reliability, and long-term drift suppression. Thus, the design principle for multiscale hybrid carbon sensors should shift from maximizing multifunctionality to achieving controlled, reproducible, and application-specific integration.

Beyond the intrinsic properties of individual carbon materials, the practical performance of flexible strain sensors is strongly influenced by device architecture, filler distribution, interfacial bonding, microstructural design, and testing conditions. Consequently, reported values of sensitivity, gauge factor, sensing range, durability, and application performance are often difficult to compare directly across different studies. To provide a clearer performance-level comparison, Table 2 summarizes representative carbon-based flexible strain sensors discussed in this review, including their material systems, device configurations, sensing mechanisms, sensitivity or gauge factor, sensing range, stability or durability, and target applications..

**Table 2:** Performance comparison of representative carbon-based flexible strain sensors for wearable and implantable applications

Carbon material system	Device structure or substrate	Sensing mechanism	Sensitivity or GF & sensing range	Stability or durability	Target application
Porous Ecoflex/MWCNT composite, PEMC [47]	Porous elastomeric capacitive pressure sensor	Capacitive	6.42 kPa⁻^1^ Pressure sensing range NR in current draft	Porous architecture improves mechanical compliance	Wearable pressure/tactile sensing
Wrinkled CNT electrodes/tape dielectric [48]	Ultrastretchable capacitive strain sensor with two wrinkled CNT electrodes	Capacitive	GF = 2.07 300% strain	High linearity and negligible hysteresis	Finger and knee motion monitoring
PVDF/CNF/PAMPS composite [65]	Hydrophilic piezoelectric nanocomposite membrane	Piezoelectric	Peak output voltage = 3.65 V Dynamic deformation sensing; exact strain range NR in current draft	β-phase PVDF promoted by PAMPS; stability data NR in current draft	High-voltage piezoelectric sensing, wearable dynamic sensing
PAM/MMT/CNT glycerol hydrogel [75]	Antifreezing CNT-based hydrogel	Piezoelectric	Operational temperature range: − 60 to 60 °C	Stable output over broad temperature range	Human activity discrimination under different intensities
CNT@Ni carbon nanotube fiber [97]	Core-sheath fiber with gradient conductivity	Piezoresistive	Sensitivity = 81.58 Maximum fracture strain = 210%	Improved strain sensing performance through gradient structure	Fiber-shaped wearable strain sensor
GNP/PDMS strain sensor [112]	Graphene nanoplatelets on PDMS substrate	Piezoresistive	GF = 320 at 0.01% strain; GF up to 4200 at 10% strain Up to 10% strain reported in current draft	Ultrahigh sensitivity	High-resolution small-strain detection
rGO/CNT brick-and-mortar structure [116]	Wrinkled rGO/CNT composite film	Piezoresistive	Designed to balance stretchability and sensitivity	Addresses stretchability-sensitivity trade-off	Wearable strain sensing
Pre-strain-assisted rGO/epoxy sensor [117]	Directionally aligned rGO/epoxy strain sensor	Piezoresistive	GF = 80.07 at 25% pre-strain 9.43-fold improvement over unstrained counterpart	Anisotropic response through directional microstructural alignment	Directional strain sensing
rGO/GR composite sensor [128]	Dip-coated rGO/graphene-based flexible sensor	Piezoresistive	Sensitivity = 15.081	Sensor performance tuned by rGO/GR ratio and dip-coating cycles	Infant sleep posture monitoring and danger warning
Graphene aerogel, GA [135]	Honeycomb/hierarchical porous graphene aerogel	Piezoresistive	Sensitivity = 1.744 kPa^−1^ Low-pressure physiological sensing	Hierarchical porous structure improves physiological signal detection	Arterial pulse monitoring
Wafer-scale 3D graphene [20]	Femtosecond-laser-patterned 3D graphene on Si substrate	Piezoresistive	GF∥ = 413 GF⊥ = 22	Anisotropic electromechanical response; enhanced durability mentioned	Flexible anisotropic strain sensing

The comparison in Table 2 indicates that high-performance carbon-based strain sensors are usually achieved through structural amplification strategies, such as microcrack engineering, wrinkled electrodes, porous conductive networks, aerogel frameworks, and hybrid filler architectures. These strategies effectively enhance electromechanical coupling and expand the detectable deformation range. However, many reported devices still emphasize peak sensitivity or gauge factor, whereas other practically important parameters, including hysteresis, response/recovery behavior, long-term drift, cyclic durability, device-to-device reproducibility, and environmental stability, are not always systematically reported. This lack of standardized performance reporting makes direct benchmarking difficult and remains a key obstacle for translating laboratory-scale carbon-based strain sensors into reliable wearable and implantable systems.

## Multifunctional in wearable and implantable systems

**Fig. 10:**
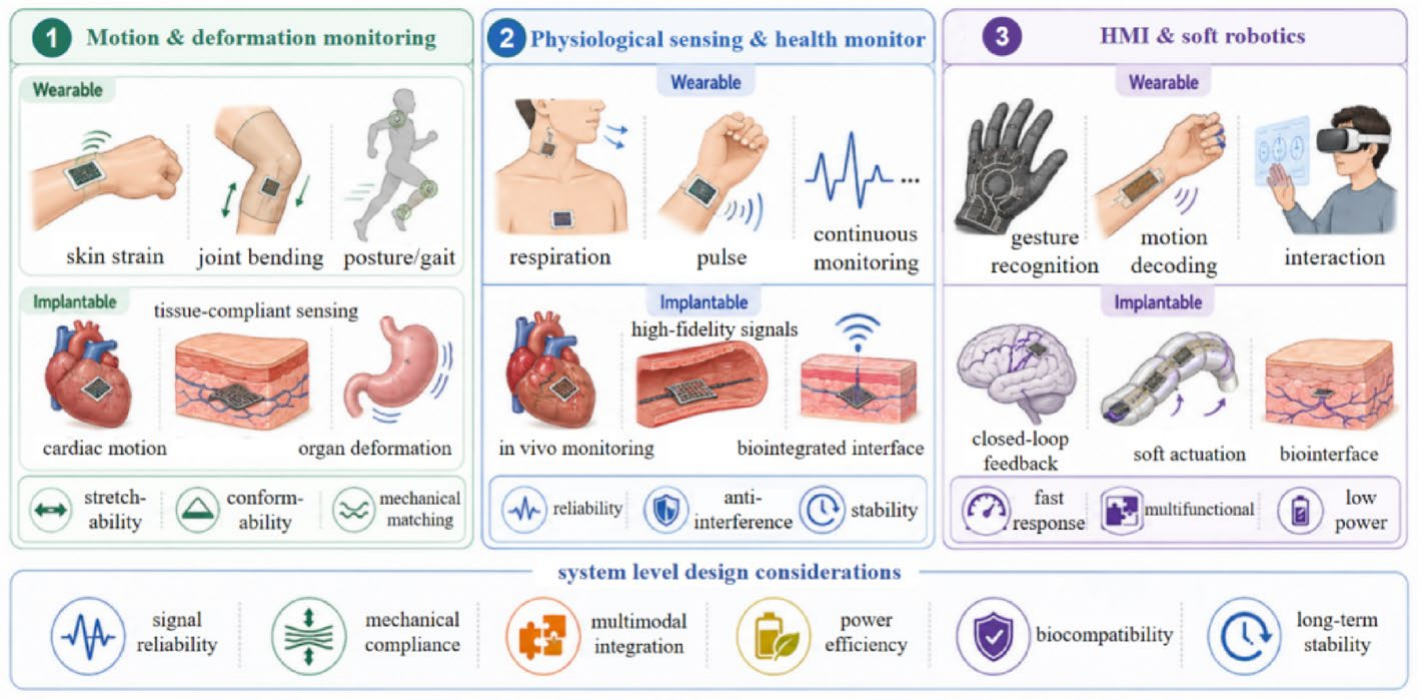
Multifunctional applications of wearable and implantable systems

The application of carbon-based flexible strain sensors is expanding from simple deformation detection toward multifunctional, biointegrated sensing systems for continuous health monitoring, human–machine interaction, and soft robotic feedback. In these scenarios, sensor performance must be evaluated not only by sensitivity or stretchability, but also by long-term stability, signal reliability, mechanical compatibility, power consumption, and integration with other sensing or actuation functions. As shown in Fig. 10, representative multifunctional applications can be broadly grouped into human motion and deformation monitoring, physiological signal sensing and health monitoring, and human–machine interfaces and soft robotics, each involving distinct wearable and implantable implementation scenarios. The figure also highlights key system-level design considerations, including signal reliability, mechanical compliance, multimodal integration, power efficiency, biocompatibility, and long-term stability. Therefore, this section summarizes representative multifunctional applications while emphasizing the distinct design requirements and unresolved challenges of wearable and implantable strain sensing systems.

### Human motion and deformation monitoring

**Fig. 11:**
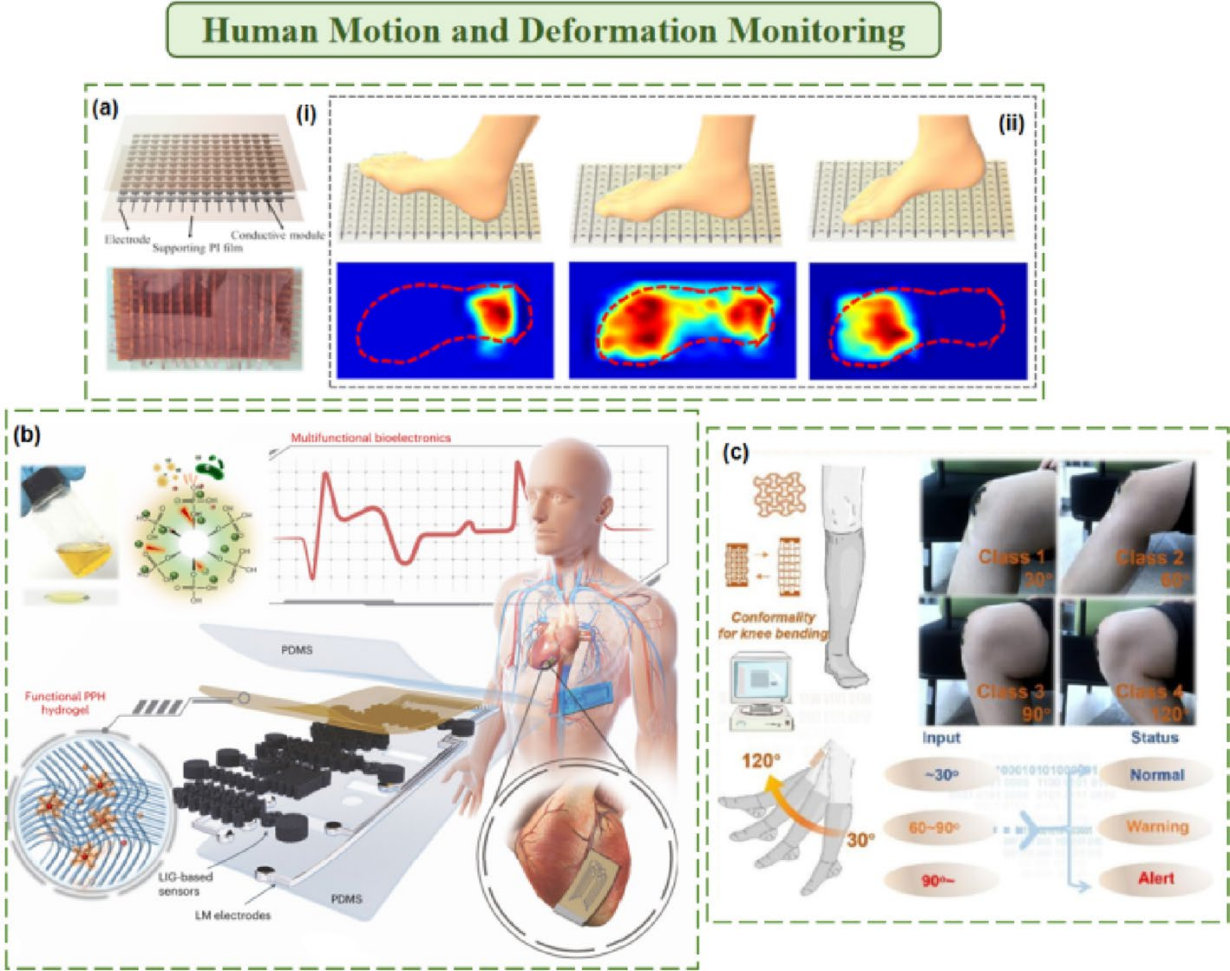
Representative applications of carbon-based flexible sensors in human motion and tissue deformation monitoring. **a** (i) Sensor array, (ii) Foot pressure distribution in different walking postures [167]. Copyright © 2022 Elsevier Ltd. **b** Thin, antibacterial and biocompatible PPH hydrogelenhanced stretchable nanocomposites for wearable and implantable bioelectronics [168]. Copyright © 2023, The Author(s), under exclusive licence to Springer Nature Limited. **c** Optical images of knee bending to detect the four bending status and schematic diagram of presupposed bending angle categorizations [169]. Copyright © 2025 Elsevier B.V

Monitoring human motion and tissue deformation represents one of the most mature and practically relevant application scenarios for carbon-based flexible strain sensors. Human activities and physiological processes generate mechanical signals over multiple length and time scales, ranging from plantar pressure, joint bending, muscle contraction, and skin stretching to periodic deformation of internal organs, such as cardiac beating, vascular pulsation, respiration-related expansion, and gastrointestinal peristalsis. Representative examples include plantar pressure mapping, knee bending recognition, and stretchable hydrogel-based interfaces for wearable and implantable bioelectronics, as shown in Fig. 11. For these applications, flexible strain sensors are expected not only to detect deformation with high sensitivity, but also to maintain mechanical compliance and signal reliability without interfering with natural body motion or tissue function.

In wearable applications, carbon-based flexible strain sensors are commonly attached to the skin or integrated into textiles to monitor finger bending, wrist motion, knee flexion, gait patterns, facial expressions, and other body movements. Representative studies based on conductive graphene/TPU foam and CNT/rGO/PDMS composites have demonstrated wide-range deformation detection, high sensitivity, and rapid response for human motion monitoring [170, 171]. However, practical motion monitoring should not be evaluated only by the number of recognizable gestures or the peak gauge factor reported under laboratory conditions. Long-term attachment stability, sweat-induced baseline drift, sensor displacement, inter-user variability, and repeated wearing/removal cycles can all substantially affect signal reliability. Therefore, wearable motion sensors should be assessed under realistic conditions, including prolonged operation, large-amplitude cyclic deformation, skin curvature variation, and motion artifacts. From a materials perspective, carbon nanotubes, graphene derivatives, carbon black, and porous carbon networks provide effective conductive pathways for deformation sensing, but their network reconstruction under repeated strain may also introduce hysteresis and irreversible drift. Thus, the key design objective for wearable motion monitoring is not simply maximizing sensitivity, but achieving a balanced combination of stretchability, mechanical robustness, comfort, low hysteresis, and reproducible signal output.

Implantable deformation monitoring imposes more stringent and fundamentally different requirements. Unlike epidermal sensors, implantable devices operate in moist, confined, and mechanically dynamic biological environments, where biofluid infiltration, tissue adhesion, inflammatory response, encapsulation failure, and mechanical mismatch may all degrade sensing performance. Carbon dot-based mechanofluorescent hydrogels and 4D-printed multifunctional hydrogels have shown the potential of soft, deformable materials for strain tracking, tissue-adaptive structures, and implantable bioengineering applications [172, 173]. Nevertheless, for monitoring cardiac motion, bladder expansion, vascular pulsation, or gastrointestinal peristalsis, the sensor must be sufficiently soft and thin to avoid constraining the target tissue, while also maintaining stable electromechanical transduction over extended periods. In this context, ultrathin films, wavy structures, soft hydrogel composites, and elastomer-encapsulated carbon networks are promising strategies for reducing tissue interference and improving conformal contact. However, implantable deformation sensing remains limited by long-term drift, sterilization compatibility, wireless data and power transmission, and the difficulty of calibrating sensor outputs after implantation. Therefore, future implantable carbon-based strain sensors should be designed around tissue-level mechanical matching, biofluid-resistant encapsulation, low-power operation, and long-term calibration stability rather than short-term sensitivity alone.

Another emerging direction is the transition from single-point strain detection to spatially resolved and multifunctional deformation mapping. Sensor arrays can provide information on deformation distribution, which is valuable for gait analysis, rehabilitation assessment, soft prosthetics, and organ-level biomechanical monitoring. However, increasing the number of sensing units also introduces new challenges, including channel-to-channel variation, wiring complexity, power consumption, and signal crosstalk. Similarly, integrating strain sensing with temperature, electrophysiological, or biochemical sensing can enrich physiological interpretation, but multimodal integration should not be regarded as a straightforward improvement. Different sensing modalities may interfere mechanically, electrically, or chemically, and their signals often require careful decoupling and calibration. Robust signal-fusion frameworks are still insufficiently developed for flexible and implantable sensor systems. Therefore, the future of human motion and tissue deformation monitoring lies not only in constructing more sensitive carbon-based materials, but also in developing reliable system-level designs that combine stable materials, artifact-resistant device structures, low-power electronics, and interpretable data-processing strategies.

### Physiological signal sensing and health monitoring

**Fig. 12:**
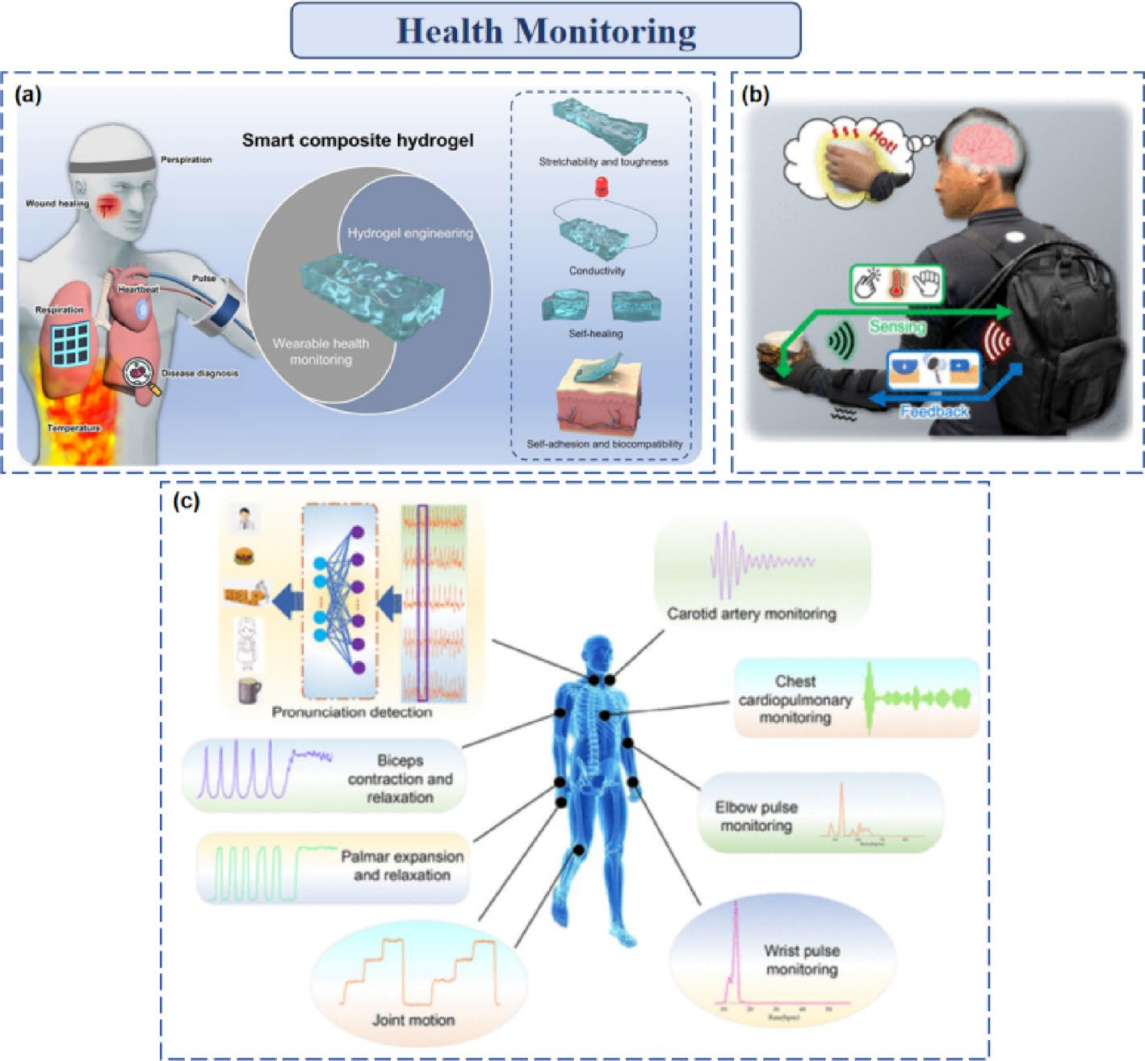
Representative applications of flexible sensing systems in physiological signal sensing and health monitoring. **a** Smart composite hydrogels for wearable health monitoring [174]. Copyright © 2023, The Author(s). **b** Multimodal sensing and feedback system for upper-limb sensory impairment assistance [175]. Copyright © 2025, The Author(s). **c** Health monitoring sensor with multiple applications [176]. Copyright © 2024, American Chemical Society

Physiological signal sensing and health monitoring represent a more demanding application scenario than macroscopic motion detection, because the target signals are usually weaker, slower, and more susceptible to interference. Typical physiological strain-related signals include pulse waves, respiratory movements, swallowing, vocal cord vibration, subtle skin deformation, cardiac contraction, vascular pulsation, and gastrointestinal peristalsis. As illustrated in Fig. 12, flexible sensing systems have been widely explored for wearable health monitoring, multimodal feedback, and multifunctional physiological signal acquisition. Compared with large-amplitude joint motion, these signals often require higher strain resolution, lower noise, better conformal contact, and more stable baseline output over prolonged monitoring periods.

Physiological signal sensing and health monitoring represent a more demanding application scenario than macroscopic motion detection, because the target signals are usually weaker, slower, and more susceptible to interference. Typical physiological strain-related signals include pulse waves, respiratory movements, swallowing, vocal cord vibration, subtle skin deformation, cardiac contraction, vascular pulsation, and gastrointestinal peristalsis. As illustrated in Fig. 8a–c, flexible sensing systems have been widely explored for wearable health monitoring, multimodal feedback, and multifunctional physiological signal acquisition [174, 177, 178]. Compared with large-amplitude joint motion, these signals often require higher strain resolution, lower noise, better conformal contact, and more stable baseline output over prolonged monitoring periods.

In wearable health monitoring, carbon-based flexible strain sensors are commonly placed on the wrist, neck, chest, abdomen, or other body surfaces to capture pulse, respiration, swallowing, and subtle muscular activities. For example, bamboo fiber/reduced graphene oxide aerogel piezoresistive sensors have been used to detect human signals such as finger movement and swallowing, demonstrating the potential of porous carbon-based architectures for wearable physiological monitoring [175]. However, high sensitivity alone does not guarantee reliable health assessment. Surface physiological signals are strongly affected by sensor placement, skin curvature, adhesion quality, sweat, temperature fluctuation, and motion artifacts. In pulse or respiration monitoring, for instance, the measured waveform reflects not only the target physiological process but also the mechanical coupling between the sensor, skin, soft tissue, and external environment. Therefore, future wearable physiological sensors should be evaluated not only by gauge factor or response time, but also by long-term baseline drift, waveform reproducibility, anti-interference capability, inter-subject variability, and performance under daily activities.

Implantable physiological sensing can provide more direct access to organ-level biomechanical information than surface-mounted devices, enabling the monitoring of cardiac motion, vascular compliance, bladder expansion, respiratory dynamics, or gastrointestinal peristalsis. Such information may support early disease warning, postoperative recovery assessment, and closed-loop therapeutic systems [76, 176]. Nevertheless, implantable physiological monitoring imposes much stricter requirements than wearable sensing. Carbon-based sensing materials must maintain stable electromechanical properties in biofluid-rich environments while avoiding tissue irritation, inflammatory responses, filler leakage, and mechanical constraint on soft organs. Long-term implantation may also induce fibrotic encapsulation, interfacial delamination, and signal attenuation, making the initial high sensitivity of a device less meaningful if calibration cannot be maintained over weeks or months. Therefore, implantable carbon-based physiological sensors should prioritize biointerface stability, encapsulation reliability, tissue-level mechanical matching, sterilization compatibility, and low-power or wireless operation.

A further challenge lies in distinguishing physiological signals from interfering mechanical and environmental stimuli. In real monitoring scenarios, respiration, body movement, pulse waves, temperature changes, and biochemical variations may occur simultaneously. Simply integrating strain, temperature, electrophysiological, or biochemical sensing units into one device does not automatically produce a reliable health-monitoring platform. Instead, multimodal integration introduces new problems, including signal crosstalk, mismatched response times, inconsistent sampling requirements, increased circuit complexity, and higher power consumption. These issues are particularly critical for implantable systems, where space, energy supply, heat generation, and long-term biocompatibility are highly constrained [179–181]. Consequently, the future development of carbon-based physiological sensing systems should shift from demonstrating individual sensing functions toward building validated, low-noise, and artifact-resistant platforms with robust signal decoupling, calibration protocols, and clinically meaningful data interpretation.

### Human–machine interfaces and soft robotics

**Fig. 13:**
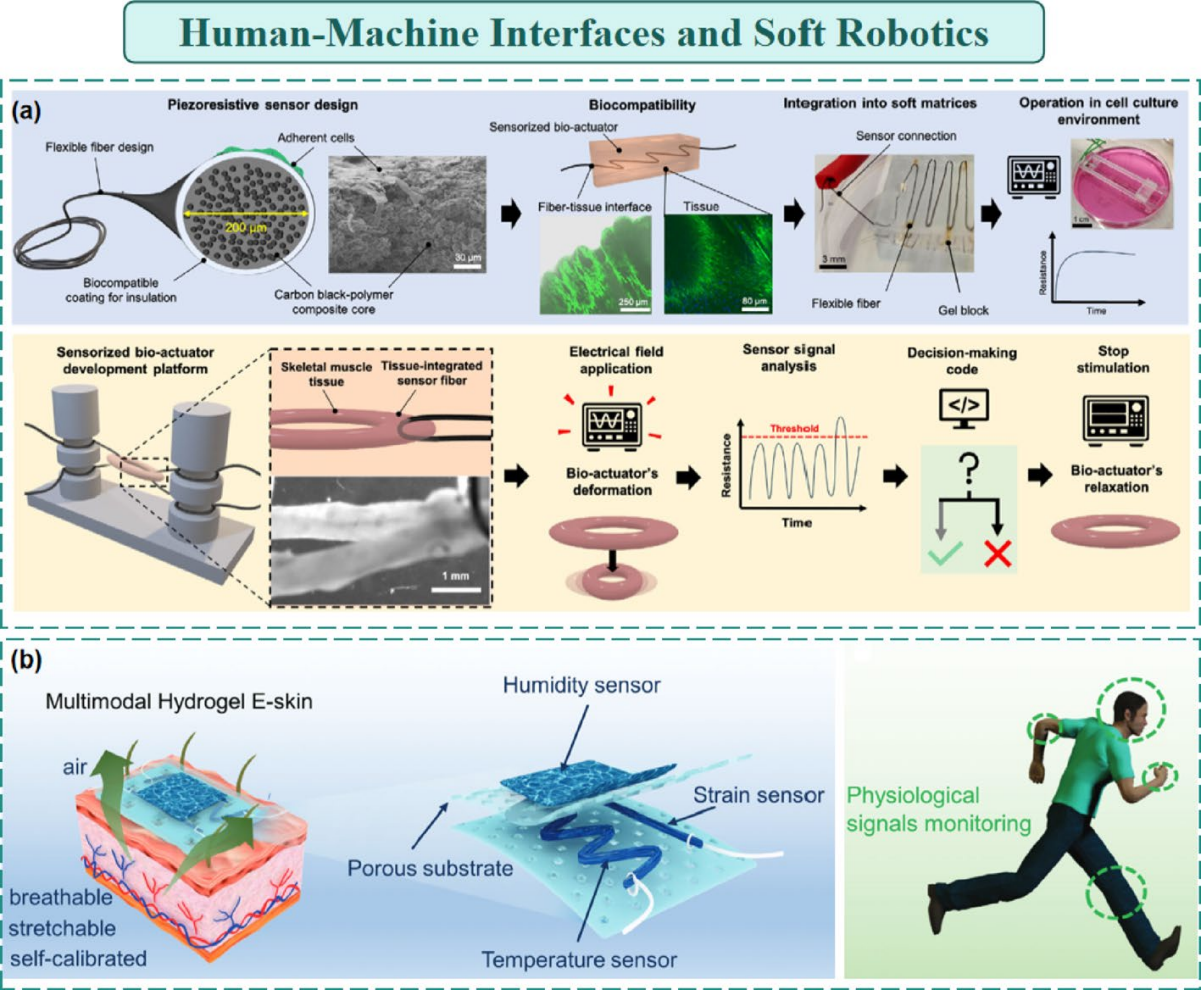
Representative applications of flexible sensing systems in human–machine interfaces, electronic skin, and soft robotics. **a** Piezoresistive sensor for proprioceptive biohybrid robots [182]. Copyright © 2024, The Author(s). **b** Structure and characteristics of the biomimetic multimodal hydrogel-based e-skin [183]. Copyright © 2024 Wiley‐VCH GmbH

Human–machine interfaces, electronic skin, and soft robotics represent advanced application scenarios for carbon-based flexible strain sensors, where mechanical deformation signals must be converted into reliable, real-time, and interpretable feedback information. Unlike simple motion monitoring, these systems require not only high sensitivity and stretchability, but also fast response, low hysteresis, repeatable output, spatial resolution, and compatibility with highly deformable biological or robotic surfaces. As shown in Fig. 13 flexible sensing systems have been explored for proprioceptive feedback in biohybrid robots and biomimetic multimodal electronic skin, highlighting their potential in closed-loop soft robotic perception and intelligent human–machine interaction. Carbon-based sensing materials, including carbon nanotubes, graphene derivatives, carbon fibers, and porous carbon networks, are attractive for these applications because they can be integrated into elastomers, textiles, hydrogels, and soft actuators to construct mechanically compliant and electrically responsive interfaces.

In wearable human–machine interfaces, flexible strain sensors are generally used to capture finger bending, wrist rotation, forearm deformation, joint movement, and muscle-related mechanical signals, which can then be translated into commands for external devices, rehabilitation systems, or interactive platforms. However, robust HMI performance depends on more than accurate gesture recognition under controlled laboratory conditions. Sensor displacement, inconsistent attachment position, sweat-induced baseline drift, user-to-user variability, fatigue-related motion changes, and repeated wearing/removal cycles can all reduce recognition reliability in practical use. Therefore, HMI-oriented carbon-based sensors should be evaluated under cross-user, long-duration, and repeated-wearing conditions, rather than relying only on short-term recognition accuracy for a limited set of predefined gestures. From a device-design perspective, maintaining signal reproducibility under natural body movement is often more important than maximizing a single sensitivity value.

In soft robotics, carbon-based flexible sensors can be integrated into soft actuators, grippers, artificial skins, or biohybrid systems to provide proprioceptive and tactile feedback. By monitoring bending, stretching, compression, and contact-induced deformation, these sensors can support closed-loop control, object manipulation, and safer human–robot interaction [184, 185]. Representative carbon-based sensing structures, such as biomimetic carbon nanofiber sensors, have demonstrated the ability to track irregular deformation in soft components, showing their potential for robotic grasping, identification, and feedback control [186]. Nevertheless, sensor integration in soft robotic systems remains challenging because the sensing layer must deform together with the actuator without restricting its motion, delaminating from the substrate, or generating nonlinear and history-dependent signals. In addition, signals generated during soft robotic operation often arise from coupled strain, pressure, bending, torsion, and contact events, making accurate signal decoupling and calibration difficult.

Electronic skin further extends flexible strain sensing from single-point deformation detection to distributed tactile perception. Pressure sensor arrays and flexible tactile interfaces are essential for mapping external force, contact location, and deformation distribution, which are key capabilities for electronic skin in robotics, artificial intelligence, and biomedical systems [187]. Carbon nanotube-based core-sheath fibers and textile-integrated sensing architectures have shown promise for constructing wearable electronic skins capable of detecting both subtle physiological signals and large body movements [188]. However, practical e-skin requires more than simply increasing the number of sensing units or integrating multiple functions. Large-area arrays introduce device-to-device variation, spatial-resolution limitations, wiring complexity, power consumption, mechanical fatigue, and encapsulation challenges [189, 190]. In particular, when strain, pressure, temperature, humidity, electrophysiological, or biochemical signals are measured simultaneously, different channels may interfere with each other mechanically, electrically, or environmentally. Therefore, multifunctional e-skin systems require rational device architecture, effective encapsulation, low-power readout circuits, and robust signal-decoupling strategies rather than simple stacking of multiple sensing modules [191].

Machine learning and pattern-recognition algorithms can assist in decoding complex deformation patterns in HMI, electronic skin, and soft robotic systems, but they should not be regarded as a universal solution to sensor-level limitations. The performance of ML-assisted interpretation strongly depends on the size, diversity, and quality of training datasets. Models trained on a small number of users, fixed sensor positions, or simplified motion patterns may fail when exposed to new users, sensor displacement, long-term signal drift, environmental changes, or deformation modes outside the training distribution [192]. Moreover, the lack of standardized benchmark datasets and evaluation protocols in flexible sensor research makes it difficult to compare algorithms across different studies [193, 194]. Therefore, future ML-assisted carbon-based sensing systems should emphasize cross-subject validation, long-term data collection, domain adaptation, uncertainty evaluation, interpretable models, and standardized datasets, rather than merely reporting high recognition accuracy under controlled experimental conditions [195].

Although wearable and implantable flexible strain sensors often share similar carbon-based conductive materials and sensing mechanisms, their design requirements are fundamentally different. Wearable sensors are mainly constrained by skin conformability, comfort, washability, motion artifacts, and repeated mechanical deformation during daily activities. In contrast, implantable sensors must operate in biofluid-rich and mechanically dynamic internal environments, where biocompatibility, tissue-level mechanical matching, encapsulation reliability, long-term signal stability, and low-power operation become decisive factors. To clarify these application-specific requirements, Table 3 compares the major design considerations for wearable and implantable carbon-based flexible strain sensors.

**Table 3:** Distinct design requirements of wearable and implantable carbon-based flexible strain sensors

Design aspect	Wearable sensors	Implantable sensors	Implications for carbon-based material design
Mechanical compliance	Need to conform to skin, joints, textiles, and curved body surfaces while tolerating repeated stretching, bending, and compression	Need tissue-level modulus matching to avoid mechanical irritation, interfacial delamination, and disturbance of native organ motion	Use soft elastomers, hydrogels, ultrathin films, wavy structures, porous scaffolds, and low-loading conductive networks
Target deformation	Large-amplitude motions such as finger bending, knee flexion, gait, facial expression, and muscle movement	Small, periodic, and often confined deformation from cardiac beating, respiration, vascular pulsation, bladder expansion, or gastrointestinal peristalsis	Wearable sensors may prioritize large stretchability and robustness; implantable sensors should prioritize low modulus, high resolution, and minimal mechanical constraint
Sensitivity requirement	Needs to cover both large joint motion and subtle physiological signals such as pulse, respiration, swallowing, or facial expression	Needs high fidelity for weak internal biomechanical signals under moist and dynamic conditions	Material design should avoid pursuing ultrahigh GF alone; sensitivity must be balanced with signal stability, strain range, and drift resistance
Signal stability	Affected by sweat, skin movement, sensor displacement, washing, temperature variation, and repeated attachment/removal	Affected by biofluid penetration, tissue reaction, fibrotic encapsulation, encapsulation failure, and long-term interfacial instability	Encapsulation, hydrophobic treatment, antifouling surface modification, and stable filler-matrix interfaces are critical
Biocompatibility	Requires skin comfort, low irritation, breathability, and safe long-term contact	Requires cytocompatibility, low immune response, stable encapsulation, sterilization compatibility, and prevention of filler leakage	Carbon fillers should be embedded in biocompatible matrices or protected by reliable encapsulation layers; exposed interfaces require careful surface modification
Power and readout	Can usually tolerate small external modules, batteries, or wireless readout units	Strongly constrained by miniaturization, heating, wireless power/data transmission, and surgical accessibility	Low-power piezoresistive readout, capacitive stability, or self-powered piezoelectric/triboelectric modes may be selected depending on application
Environmental robustness	Needs resistance to humidity, sweat, washing, friction, and daily mechanical disturbance	Needs stable function in biofluids, ionic environments, enzymatic conditions, and long-term mechanical cycling	Stable carbon dispersion, strong interfacial bonding, low-drift conductive networks, and encapsulation reliability are essential
Device format	Patches, textiles, gloves, belts, sleeves, flexible films, electronic skin, and soft robotic interfaces	Ultrathin films, soft hydrogel interfaces, organ-mounted sensors, bioelectronic interfaces, flexible electrodes, and implantable closed-loop systems	Wearable formats can emphasize comfort and scalability; implantable formats require miniaturization, soft integration, and surgical compatibility
Manufacturing scalability	Requires low-cost, large-area, reproducible, and washable fabrication	Requires reproducible microfabrication, sterilization compatibility, packaging reliability, and regulatory feasibility	Wet spinning, coating, printing, laser writing, and template-assisted fabrication should be evaluated not only by performance but also by batch consistency
Translation bottleneck	User comfort, motion artifacts, device-to-device consistency, calibration stability, and long-term wearability	Long-term safety, drift-free operation, encapsulation, tissue adhesion, immune response, and clinical validation	Future studies should report standardized durability, hysteresis, drift, environmental stability, and in vivo reliability metrics

This comparison highlights that wearable and implantable strain sensors should not be evaluated using identical criteria. For wearable systems, high stretchability, comfort, scalable fabrication, and robustness against sweat, friction, and repeated attachment are often prioritized. For implantable systems, however, the central challenge shifts from short-term sensitivity enhancement to long-term biointerface reliability, mechanical matching with soft tissues, suppression of signal drift, and safe operation in complex physiological environments. Therefore, future carbon-based sensor design should adopt an application-oriented strategy, in which material selection, device architecture, encapsulation, and signal readout are optimized according to the specific biological interface and monitoring scenario.

## Challenges and future perspectives

Despite rapid progress, the development of carbon-based flexible strain sensors is entering a stage in which the major bottleneck is no longer the demonstration of high sensitivity, stretchability, or multifunctionality alone, but the translation of these devices into reliable, interpretable, standardized, and clinically meaningful wearable and implantable systems [196–198]. Recent studies increasingly combine flexible sensing, multimodal signal acquisition, and machine-learning-assisted modeling for motion analysis, physiological monitoring, human–machine interaction, and disease-related pattern classification [4]. However, this apparent prosperity also conceals several unresolved problems, including limited device reliability, insufficient standardization, weak model generalizability, inadequate interpretability, multimodal signal interference, and the lack of clinically validated signal-fusion frameworks.

A central materials-level challenge remains the trade-off among sensitivity, working range, linearity, and long-term stability. Many piezoresistive carbon-based strain sensors achieve high gauge factors by operating near the percolation threshold, enhancing tunneling effects, introducing microcrack structures, or constructing porous conductive networks. Although these strategies can amplify electrical responses, they may also increase nonlinearity, hysteresis, baseline drift, and irreversible reconstruction of conductive pathways during repeated deformation [196]. Similarly, three-dimensional carbon aerogels, foams, and hybrid networks can improve stretchability and deformation tolerance, but their signals may depend on pore collapse, interfacial sliding, crack propagation, or skeleton-contact variation, which are vulnerable to fatigue under long-term cyclic loading. Therefore, future studies should move beyond reporting peak sensitivity or maximum gauge factor alone and should systematically evaluate complete performance profiles, including sensing range, linearity, hysteresis, response/recovery time, drift, cyclic repeatability, environmental stability, and device-to-device variation [197].

Long-term stability in realistic biological environments is another critical challenge. Wearable sensors are exposed to sweat, humidity, temperature fluctuation, skin motion, mechanical abrasion, and repeated attachment–detachment cycles, while implantable sensors must function in biofluid-rich, chemically complex, and mechanically dynamic environments [197, 198]. Biofluid penetration, encapsulation failure, filler leakage, interfacial delamination, protein adsorption, inflammatory responses, and foreign-body reactions may progressively degrade both sensing performance and biological safety. For implantable applications in particular, high initial sensitivity is insufficient; stable, calibrated, and biocompatible signal output over extended periods is more important. Future designs should therefore emphasize tissue-level mechanical matching, robust encapsulation, anti-biofouling interfaces, sterilization compatibility, low-power operation, and long-term in vivo validation [20, 118].

Standardization is also a major unresolved issue in flexible wearable and implantable strain sensing. At present, many studies use different testing protocols, including strain range, strain rate, loading mode, cycle number, substrate geometry, device dimensions, electrode configuration, and environmental conditions, making direct comparison across reports difficult [196, 197]. More importantly, raw sensing data, preprocessing methods, device metadata, and subject information are often insufficiently reported, which prevents datasets from being reused or merged across laboratories. This lack of standardized, shareable datasets is particularly problematic for disease-related modeling, where robust classification requires data from diverse subjects, disease stages, sensor positions, activity states, and long-term monitoring conditions [199–201]. Future research should establish community-level benchmark protocols and open datasets that include not only sensor signals but also calibration procedures, environmental conditions, subject-level metadata, annotation standards, preprocessing pipelines, and clinically relevant ground truth labels.

Machine learning and pattern recognition can assist in interpreting complex strain signals for human–machine interfaces, rehabilitation monitoring, soft robotics, and health-related classification, but they should not be presented as universal solutions [199]. Their performance strongly depends on the size, diversity, quality, and labeling accuracy of training datasets. Models trained using limited subjects, fixed sensor positions, controlled motion patterns, or short-term measurements may fail when applied to new users, different disease populations, altered sensor placements, unseen deformation modes, or long-term operating conditions [200, 202]. In disease analysis, this problem becomes even more pronounced because physiological signals are affected by age, sex, body shape, comorbidities, medication, disease stage, daily activity, and environmental factors. High classification accuracy obtained under laboratory conditions may therefore reflect dataset bias, subject-specific features, or experimental artifacts rather than disease-specific mechanisms.

The interpretability of ML-assisted flexible sensor systems is also insufficient. Common approaches such as ablation analysis, feature ranking, dimensionality reduction, or attention visualization may indicate which signals contribute to classification, but they do not necessarily explain the physiological or pathological meaning of these signals [201]. For clinically relevant applications, models should not only output a disease category, motion label, or risk score, but should also clarify whether the decision is based on meaningful biomechanical, electrophysiological, biochemical, or behavioral features. Future ML-assisted systems should therefore incorporate cross-subject and cross-device validation, external test cohorts, uncertainty estimation, domain adaptation, physics- or physiology-informed modeling, interpretable feature representations, and failure detection mechanisms. Instead of merely reporting high classification accuracy, future studies should report robustness under distribution shift, calibration error, confidence reliability, and performance degradation when sensors age, drift, or are repositioned [203].

Multimodal integration requires even more critical consideration. Integrating strain sensing with temperature, electrophysiological, tactile, humidity, pressure, or biochemical monitoring can provide richer information for health monitoring and disease classification, but the key challenge is no longer simply combining multiple sensing units [204, 205]. Different diseases may be reflected through different combinations of mechanical, electrical, thermal, and biochemical features. Therefore, multimodal systems must determine which modality is truly informative for a given application, how different modalities interact, and whether a modality has become unreliable or clinically irrelevant under certain conditions. Without such evaluation, multimodal sensing may increase system complexity without improving diagnostic value.

Signal crosstalk is one of the most important engineering barriers in multimodal flexible sensors. Resistance changes attributed to strain may also arise from temperature variation, humidity, sweat absorption, electrode polarization, ion diffusion, biochemical reactions, or interfacial instability [206, 207]. Electrophysiological signals can be distorted by motion artifacts, while biochemical signals can be affected by sweat rate, evaporation, skin contamination, and sensor aging. If these effects are not effectively decoupled, multimodal devices may generate misleading outputs despite containing multiple sensing channels. Future multifunctional carbon-based sensors should therefore employ rational device architectures, spatially separated or orthogonal sensing pathways, material-level selectivity, compensation channels, self-calibration strategies, low-power readout circuits, and robust signal-fusion algorithms capable of identifying corrupted, missing, or low-confidence modalities [203, 208].

Power consumption, wireless communication, data security, and system integration are additional barriers to practical use. Implantable and long-term wearable systems cannot rely on bulky power supplies or frequent recalibration. Multimodal sensing and machine-learning inference further increase energy demand, especially when continuous monitoring, wireless transmission, and real-time classification are required [196, 203]. Future systems should integrate low-power sensing mechanisms, edge computing, compressed data acquisition, energy harvesting, adaptive sampling, and privacy-preserving data processing. For disease monitoring, clinically useful systems should prioritize stable longitudinal trends, early warning capability, and interpretable risk assessment rather than isolated short-term classification results.

Scalable manufacturing and reproducibility must also be addressed. Many high-performance carbon-based sensors rely on finely tuned microstructures, such as aligned nanotube networks, wrinkled graphene films, porous aerogels, crack-engineered layers, or multiscale hybrid architectures. Although effective in laboratory demonstrations, these structures may suffer from limited reproducibility, batch-to-batch variation, and sensitivity to processing conditions [197]. Future work should focus on scalable fabrication, quality-control standards, reproducible microstructure formation, manufacturing-compatible device architectures, and statistical reporting across multiple devices and batches. For implantable sensors, cytocompatibility, inflammatory response, encapsulation reliability, sterilization tolerance, and long-term in vivo signal stability should also be evaluated systematically [209, 210].

Future development should shift from maximizing isolated performance metrics toward reliability-centered, application-specific, and system-integrated design. Carbon materials offer structural tunability, electrical versatility, and compatibility with soft substrates, making them promising for next-generation wearable and implantable sensing platforms. However, their practical impact will depend on solving challenges related to long-term stability, biointerface reliability, standardized datasets, multimodal signal decoupling, power management, interpretable modeling, and reproducible manufacturing. Through rational material design, standardized evaluation, robust encapsulation, clinically relevant validation, and trustworthy signal-fusion frameworks, carbon-enabled flexible strain sensors can move closer to practical applications in personalized healthcare, disease monitoring, rehabilitation assessment, intelligent human–machine interaction, soft robotics, and biointegrated medical systems.

## Data Availability

No datasets were generated or analysed during the current study.
